# Evaluation of a Dietary Grape Extract on Oxidative Status, Intestinal Morphology, Plasma Acute-Phase Proteins and Inflammation Parameters of Weaning Piglets at Various Points of Time

**DOI:** 10.3390/antiox11081428

**Published:** 2022-07-22

**Authors:** Emina Rajković, Christiane Schwarz, Stefan Bruno Kapsamer, Karl Schedle, Nicole Reisinger, Caroline Emsenhuber, Vladimira Ocelova, Nataliya Roth, Dörte Frieten, Georg Dusel, Martin Gierus

**Affiliations:** 1 FFoQSI GmbH—Austrian Competence Centre for Feed and Food Quality, Safety and Innovation, 3430 Tulln, Austria; emina.rajkovic@ffoqsi.at; 2 Institute of Animal Nutrition, Livestock Products, and Nutrition Physiology (TTE), Department of Agrobiotechnology, IFA-Tulln, University of Natural Resources and Life Sciences, 1190 Vienna, Austria; s.kapsamer@gmx.at (S.B.K.); karl.schedle@boku.ac.at (K.S.); martin.gierus@boku.ac.at (M.G.); 3 DSM—BIOMIN Research Center, Technopark 1, 3430 Tulln, Austria; nicole.reisinger@dsm.com (N.R.); caroline.emsenhuber@dsm.com (C.E.); vladimira.ocelova@dsm.com (V.O.); nataliya.roth@dsm.com (N.R.); 4 Animal Nutrition, Department Life Sciences, University of Applied Sciences Bingen, 55411 Bingen am Rhein, Germany; d.frieten@th-bingen.de (D.F.); g.dusel@th-bingen.de (G.D.)

**Keywords:** grape polyphenols, weaning piglets, antibiotic treatment, gene expression, antioxidant capacity, antibiotic alternatives, phytogenics, intestinal morphology, acute-phase proteins, *Vitis vinifera*

## Abstract

Reports of the underlying mechanisms of dietary grape extract (GE) in overcoming weaning challenges in piglets have been partly inconsistent. Furthermore, evaluations of the effects of GE at weaning in comparison to those of widely used therapeutic antibiotics have been scarce. To explore the mode of action of GE in selected tissues and plasma, we evaluated gut morphology, antioxidant and inflammation indices. Accordingly, 180 weaning piglets were allocated to three treatment groups: negative control (NC), NC and antibiotic treatment for the first 5 days of the trial (positive control, PC), and NC and GE (entire trial). The villus surface was positively affected by GE and PC on day 27/28 of the trial in the jejunum and on day 55/56 of the trial in the ileum. In the colon, NC tended (*p* < 0.10) to increase crypt parameters compared to PC on day 55/56. The PC group tended (*p* < 0.10) to increase catalase activity in the ileum and decrease Cu/Zn-SOD activity in the jejunum, both compared to NC. There were no additional effects on antioxidant measurements of tissue and plasma, tissue gene expression, or plasma acute-phase proteins. In conclusion, GE supplementation beneficially affected the villus surface of the small intestine. However, these changes were not linked to the antioxidant and anti-inflammatory properties of GE.

## 1. Introduction

Commercial weaning takes place during a time of the still-incomplete maturation of a piglet’s intestine [1]. This includes the morphological and functional maturation of the intestinal immune and enteric nervous systems [1,2,3]. Stressful early-life events, e.g., weaning, may disrupt gut maturation and have long-term consequences for animal health and productivity [4].

Biotic and abiotic stressors during weaning modify antioxidant capacity through an excessive accumulation of reactive oxygen species (ROS) [5], resulting in oxidative stress [6]. Under physiological circumstances, antioxidants can balance the amount of ROS [7]. At weaning, intestinal disorders and inflammation often occur and launch a cascade of defence and anti-inflammatory reactions [8,9]. To deal with the challenge of pathogens, the innate immune system starts local and systemic acute-phase inflammatory responses [10]. This begins with the production of proinflammatory proteins (e.g., cytokines) [11], and the cell consequently generates ROS [12]. ROS play a key role in the progression of inflammation processes and the tissue repair of the intestine [13], but a persistent excessive accumulation of ROS can damage all cellular structures including the intestinal epithelium [14].

The intestinal epithelium is the body’s largest mucosal surface, a restrictive barrier between the environment and the host [15]. The integrity of this barrier is essential to prevent pathogenic proliferation and chronic inflammatory processes in the intestine [16]. However, epithelial mucosal cells are not a simple physical barrier, and they play a notable role in a variety of immune functions [9,17]. Excessive ROS target epithelial cells in the first line by attacking the epithelial cell wall and cell biomolecules (proteins, lipids, and genetic material) [18]. Cell damage is induced, e.g., in oxidative processes on the cell membrane phospholipids driven by ROS (lipid peroxidation) [12]. Damage to the intestinal epithelium can compromise a release of protective substances such as antimicrobial proteins and mucin, which impairs epithelial barrier function [9,12,19] with consequences for intestinal morphology [4].

Since it is exposed to different antigens (dietary antigens and pathogens), as well as to commensal bacteria, at weaning, a piglet’s intestine aims to maintain homeostasis between all these challenges and the host immune system [17]. Animals that fail to adapt to this disrupted functionality of the intestinal epithelium and redox balance develop diarrhoea [4,12,20], followed by losses in nutrient digestibility and poor animal performance.

All these critical events during weaning lead to an overuse of antibiotics, which tackle pathogens causing diarrhoea and enteric disease incidence before leading to negative impacts on performance [21,22]. Besides the concerns of rising antimicrobial resistance worldwide [23], microbiome disturbances and oxidant–antioxidant imbalances have also been reported to be induced by a supply of bactericidal antibiotics [24]. Furthermore, disorders in the redox balance of an organism due to the inadequate removal of ROS by endogenous antioxidants may even require exogenous sources of antioxidants (supplied through the diet) [25].

Increasing evidence supports the applicability of grape polyphenols at weaning in the context of attenuating enteric disease outbreaks [26,27], which may generally reduce the need for routine antibiotic treatments at weaning [28]. In addition to antimicrobial properties [29], polyphenols have a high antioxidant [30,31] and anti-inflammatory potential [32] that may aid in overcoming the issues of weaning and promoting gut maturation [8]. Polyphenols (e.g., anthocyanins, flavonoids, and tannins) are the main antioxidant compounds present in grape by-products [33]. The antioxidant capacity of phenolic compounds is based on the maintenance of ROS homeostasis (via free radical scavenging, as substrate for attack by superoxide) and a decrease in lipid oxidation [34,35], which rest on the presence of electron-donating phenolic groups in their structure [36]. However, polyphenols also seem to affect cell-signalling pathways and gene expression [37].

Recently published work from our research group [38] confirmed the potential of polyphenols from grape extract (GE) in partly enhancing the nutrient digestibility of weaning piglets over the level of a positive control (five days antibiotic of treatment post weaning). We expected to observe an influence of GE on the microbial fermentation products in the intestines; however, analysed concentrations of microbial metabolites could not explain the mechanism of improved digestibility. Furthermore, we did not observe any advantages of GE inclusion on animal performance compared to the negative control or antibiotic treatment (positive control). Therefore, we assumed that the enhancement in nutrient digestibility may have been related to positive changes in intestinal morphology and/or antioxidant and immunological status of the weaning piglets. We hypothesized that the antioxidant capacity of GE can approximate potential positive effects of antibiotic treatment at weaning. Such pathways may indirectly prevent compromising alterations in intestinal morphology, as well as their effects on related gene expression, and eventually affect the gut barrier and absorptive capacity. We also hypothesized that antioxidant and stress-related measurements (acute-phase proteins) from plasma correlate with weaning and that grape polyphenols have potential to positively affect these measurements as the piglets age. The objective of this study was to look for further mechanisms that could explain the results from our previous study, as well as to explain the mode of action of dietary GE supplementation on pathways involved in antioxidative and anti-inflammatory responses.

## 2. Materials and Methods

The experimental procedures in this study were conducted in strict accordance with the German Animal Welfare Act and were approved by the relevant Department for Animal Welfare Affairs (Landesuntersuchungsamt Rheinland-Pfalz, Koblenz, Germany; registration no. 23 177-07/G 13-20-069).

### 2.1. Piglets, Diets and Housing

A trial with 180 weaning piglets with an average initial body weight (BW) of 6.9 ± 0.1 kg was performed. Piglets (DanBred × Piétrain) were individually identified using ear tags, weighed, and selected two days before start of the trial. Piglets were divided according to BW and sex in three treatment groups (each with 6 pens containing five castrated males and 6 pens containing five female piglets) at weaning (23 ± 1 day of age) and fed the experimental diets for 8 weeks, as follows: (1) a corn-based control diet (negative control, NC); (2) NC and amoxicillin trihydrate (20 mg amoxicillin/kg BW every 12 h; amoxicillin-trihydrate 100, Bela-Pharm GmbH & Co. KG, Vechta, Germany) for the first 5 days of the trial (according to manufacturer’s recommendation indicated for treatment of enteric infections in pigs)) (positive control, PC); (3) NC and grape extract (150 g/t of feed) (GE) for the whole trial duration. Grape extracts (dried extract from dried grapes *Vitis vinifera*), with an analysed content of total polyphenols > 40%, were provided by Biomin Holding GmbH (Getzersdorf, Austria). The diets were formulated to meet or exceed the nutrient requirements for piglets according to the German Society for Nutrition Physiology [39]. Animals received diets in ground form in the starter (day 1–day 13) and grower (day 14–day 56) phases. The GE powder was included in a maize premix and added to the basal diet at 0.5%. In this respect, NC and PC received 0.5% pure ground maize in the starter and grower phases. More details about animals, diets and housing were presented by Rajković et al. [38].

### 2.2. Sample Collection and Storage

Two days before the trial start (day 0) and following the antibiotic treatment (day 6 of the trial), blood samples of all animals (*n* = 180) were drawn by the puncture of cranial vena cava and collected in EDTA tubes (S-Monovette^®^ EDTA, 4.9 ml K3E, Sarstedt AG & Co. KG, Nümbrecht, Germany). During two consecutive days (day 27/28 and day 55/56), one piglet per pen (*n* = 36 per sampling point; *n* = 72 in total) with the median BW of the pen was selected for euthanasia and subsequent sampling. Before sedation for euthanasia, blood samples were drawn from the external jugular vein and collected in EDTA tubes (Kabavette EDTA, 7.5 mL, KABE Labortechnik GmbH, Nümbrecht-Elsenroth, Germany).

At every sampling point, the blood samples were stored cool at 2–8 °C immediately after the sampling. Plasma was obtained by the centrifugation of the blood samples, within 30 min after the sampling at 2000× *g* for 20 min. The acquired plasma was transported on dry ice and stored at −80 °C for further analyses of antioxidant capacity and acute-phase proteins.

At day 27/28 and day 55/56, the piglets were euthanized for tissue sampling. For this purpose, the abdominal cavity was opened immediately after euthanasia (as described by Rajković et al. [38]) and the gastrointestinal tract was ligated at the oesophageal region of the stomach and at rectum. Subsequently, the gastrointestinal tract was removed from the abdomen and prepared for sampling. The sampling of the tissue (~3 cm) for respective analyses from the jejunum, ileum and colon was performed for every animal in the same sequence from distal to proximal. Jejunum samples were excised and collected ~35 cm and ~50 cm distal from the Plica duodenocolica on day 27/28 and day 55/56, respectively. The distal end of the ileum was intersected ~2 cm proximal to the ileocecal valve and sampled. Afterwards, the colon was identified at the flexura centralis and respective samples were collected. Liver samples (~2 × 2 cm) were collected from the left medial lobe within 5 min after the ligation and removal of the gastrointestinal tract from the abdominal cavity.

**Figure 1:**
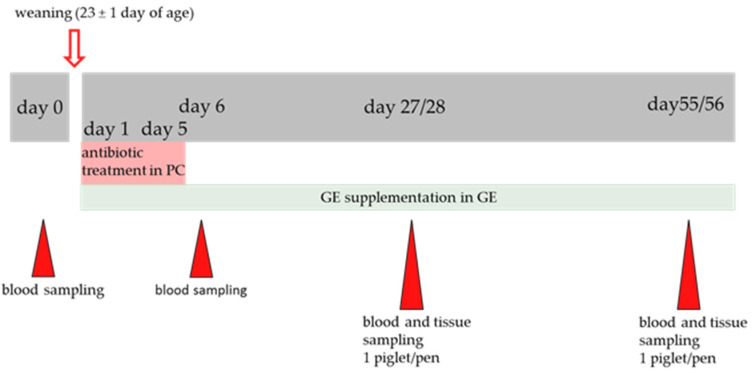
Study design and sampling overview. PC = positive control; GE = grape extract.

Segments of the gut (~2 cm) sampled for intestinal morphology measurements (as carefully as possible in order to prevent tissue damage) were flushed with ice-cold PBS (phosphate-buffered saline) and fixed in 4% formaldehyde for 48 h at room temperature. All tissue samples (jejunum, ileum and liver; ~2 cm) for the determination of antioxidant capacity were flushed with a physiological NaCl solution, rapidly frozen on dry ice, and stored at −80 °C for further analyses. For gene expression analyses, tissue samples (~1 cm) of the mid-jejunum, mid-ileum and liver were collected and placed into an Invitrogen RNAlater Stabilization Solution (Thermo Scientific, Waltham, MA, USA). The samples were stored overnight at 4 °C, transported on dry ice, and thereafter stored at −80 °C until subsequent RNA isolation. An overview of the study design and sample collection is shown in Figure 1.

### 2.3. Analyses

#### 2.3.1. Morphology of Ileum, Jejunum and Colon

After 48 h in 4% formaldehyde, gut sections of ~3 mm were cut for tissue embedding cassettes and stored in 70% ethanol for 5 days. Afterwards, the samples were embedded in paraffin wax blocks. Sections of 5 µm were processed using a microtome (Leica RM2255, Leica Biosystems GmbH, Nussloch, Germany), mounted on glass slides, and stained (Leica Auto-Stainer XL ST5010, Leica Biosystems GmbH, Nussloch, Germany) following the standard protocol for Alcian blue periodic acid–Schiff (AB–PAS) reaction. The measurements for intestinal morphology were taken using a light microscope (Leica DM 6000 B, Leica, Darmstadt, Germany) and the image processing software Leica Application Suite 4.13 (Leica Application Suite 4.13, Darmstadt, Germany). All examined morphometric indices were made on six well-orientated villi and crypts. Besides length, width, and area measurements of villi and crypts, tunica muscularis thickness (circular and longitudinal muscle layers together) was determined in six randomly selected points. The villus height-to-crypt depth ratio (VC ratio) was also calculated. Goblet cells were counted in six villi and crypts.

#### 2.3.2. Antioxidant Measurements of Liver, Jejunum and Ileum Tissue

All analyses were performed at least in duplicate. For the determination of antioxidant enzyme activity, frozen intestinal (jejunal and ileal) and liver tissue samples (~1 g and 0.5 g, respectively) were homogenized in cold (~+4 °C) potassium phosphate buffer (50 mmol/L of K_2_HPO_4_, 50 mmol/L of KH_2_PO_4_, and 1 mmol/L of EDTA) using an Ultra Turrax T25 homogenizer (IKA^®^ Werke GmbH und Co. KG, Staufen, Germany). We produced 1:5 and 1:10 homogenates for intestinal and liver samples, respectively. Triton X-100 was then added to the homogenates (for an end concentration of 1% in tissue homogenates), mixed thoroughly, and incubated on ice for 10 min. Following centrifugation at +4 °C and 2572× *g* for 10 min (5810R, Eppendorf, Wesseling-Berzdorf, Germany), the supernatants were then processed for the determination of antioxidant enzyme activity: glutathione peroxidase (GPx) [40], total superoxide dismutase (SOD), Mn-dependent SOD and Cu/Zn-SOD [41], and CAT (catalase) [42]. The buffer for tissue homogenization for GPx activity determination contained 1 mmol/L of NaN_3_. The results of the enzyme activities (U/mL) were divided by the protein concentration of the sample (expressed in mg/mL) and analysed by applying the Bradford method [43] to obtain enzyme activities expressed in U/mg or U/g of protein.

The analyses of the concentrations of thiobarbituric acid-reactive substances (TBARS) in the tissue were performed using a modified method of Ohkawa et al. [44]. First, tissue homogenates (1:5) from the jejunum and ileum were prepared. Frozen tissue aliquots (~0.5 g) or standard solution (MDA, malonaldehyde-bis-(diethylacetal) or 1,1,3,3-tetraethoxypropane; Sigma Aldrich, Steinheim, Germany) were immediately merged with 2 mL of ice-cold 1.15% KCl buffer including 60 µL of 0.3% butylated hydroxytolene in ethanol (BHT). The content was subsequently homogenized for 60 s (Ultra Turrax T25 IKA^®^ Werke GmbH und Co. KG, Staufen, Germany). For more concentrated samples (liver), 1:10 tissue homogenates using 4.5 mL of 1.15% KCl and 75 µL of 0.3% BHT were made. A combination of 0.2 mL of 8.1% sodium dodecyl sulphate, 1.5 mL of 20% acetic acid, 1.5 mL of 0.8% thiobarbituric acid and 0.8 mL of the homogenates/standard/blank was thoroughly mixed and incubated at 98.5–100 °C for 1 h (Techne^®^ Dri-Block^®^, Staffordshire, UK). After 15 min at room temperature, the addition of 2.5 mL of butanol (5 ml for liver samples) and a thorough mixing, the tubes were centrifuged at +4 °C and 3215× *g* for 10 min (5810R, Eppendorf, Wesseling-Berzdorf, Germany). After a subsequent 5 min at room temperature, the absorption of the upper layer was spectrophotometrically measured at 532 nm (Hitachi, U-5100, Tokyo, Japan). Values are expressed as µmol malondialdehyde equivalents per kg of wet tissue.

The DPPH (2,2-diphenyl-1-picrylhydrazyl) radical-scavenging activity in the tissue was evaluated following the procedure described by Brand-Williams et al. [45] with minor modifications. For this purpose, the homogenates produced for the determination of GPx-enzyme activity were used. Following, 30 µL of the homogenates or standard solution (Trolox, Sigma Aldrich, Steinheim, Germany) was added to a 2.97 mL of a diluted DPPH solution (absorbance of 0.75–0.80 at 515 nm), vigorously mixed, and incubated for 60 min at room temperature in the dark. Subsequently, the absorbance of the mixture was measured at 517 nm using a spectrophotometer (Hitachi, U-5100, Tokyo, Japan). The lower absorbance of the reaction mixture indicated higher free radical-scavenging activity. First, the absorbance of the sample/standard and blank (potassium phosphate buffer) was used to calculate the relative density following the equation:

relative density = (absorbance blank- absorbance sample/standard) / (absorbance blank)

The DPPH radical-scavenging values were then determined from the calibration curve. The values are expressed as µmol Trolox equivalents per g of wet tissue.

The homogenates produced for the determination of SOD/CAT-enzyme activity were used to measure the antioxidant capacity by the ABTS (2,2-azinobis (3-ethilenzotiazolin)-6-sulfonate) method, following the procedure described by Re et al. [46] with minor modifications. After the addition of 30 µL of the homogenate or standard solution (Trolox, Sigma Aldrich, Steinheim, Germany) to 2.97 mL of a diluted ABTS solution (7 mM ABTS stock solution with 2.45 mM potassium persulfate diluted with 50% ethanol to an absorbance of 0.75–0.80 at 734 nm), the mixture was thoroughly shaken and incubated in the dark at room temperature for 15 min (sample homogenates) or 30 min (standard solution). The content was mixed with a spatula at the beginning and the end of the incubation time. Finally, absorbance measurements were taken spectrophotometrically (Hitachi, U-5100, Tokyo, Japan) at 734 nm. The calculation of relative density was conducted as described for the DPPH method. The scavenging activity in the ABTS method was determined from the calibration curve. Values are expressed as µmol Trolox equivalents per g of wet tissue.

#### 2.3.3. Blood Parameters

##### Blood Antioxidant Status

Superoxide dismutase (SOD) and malondialdehyde (MDA) were measured with a commercially available kit (Cayman Chemical, Ann Arbor, MI, USA). Assays were performed according to the manufacturer’s manual. These analyses were performed on four sampling points of the trial (day 0, day 6, day 27/28, and day 55/56).

##### Acute-Phase Proteins as Inflammatory Parameter

The plasma concentration of the acute-phase proteins haptoglobin was measured with a commercially available ELISA kit (Life diagnostics, West Chester, PA, USA). Another acute-phase protein pigMAP (major acute-phase protein) was also measured in plasma with a commercially available ELISA kit (Acuvet Biotech, Zaragoza, Spain). ELISAs were performed according to the manufacturer’s instructions. Haptoglobin and PigMAP were analysed on three sampling points of the trial (day 6, day 27/28, and day 55/56).

#### 2.3.4. Gene Expression Analyses of Liver, Jejunum and Ileum Tissue

RNA was extracted from 30 to 50 mg of the tissue using the RNeasy^®^ Plus Mini Kit (Qiagen GmbH, Hilden, Germany). Isolated RNA samples were sent on dry ice to Qiagen, where the measurements of the concentration and quality of RNA, cDNA synthesis, and RT-qPCR were conducted. Threshold cycle (Ct) values for genes were provided and used for data analysis. RNA Integrity Number (RIN) values were above 7, with the exception of three samples (values > 6). The 2ΔΔCt method was used to determine gene expression [47] as described elsewhere [48].

Beta-actin (ACTB), beta-2-microglobulin (B2M) and glyceraldehyde-3-phosphate dehydrogenase (GAPDH) were used as reference genes for normalization. A list of the reference and selected genes is provided in Table 1.

#### 2.3.5. Statistical Analysis

All parameters were analysed using the MIXED procedure of SAS 9.4 (SAS Institute, Cary, NC, USA), with diet, sex, and the sampling day as fixed effects, as well as the inclusion of the interaction of all effects. The experimental unit was the piglet. For analyses performed in the tissues, the main effect of the sampling days comprised two levels (day 27/28 and day 55/56), whereas blood parameters were analysed on the respective three or four sampling days (day 0 = two days before the trial start; day 6, day 27/28, and day 55/56 of the trial). Using the data from four sampling days, piglet blood parameters were analysed as repeated measures using the MIXED procedure.

The applied model was:

Y_ijkl_ = µ + α_i_ + β_j_ + δ_k_ + (αβ)_ij_ + (αδ)_ik_ + (βδ)_jk_ + (αβδ)_ijk_ + ε_ijkl_

where Y_ijkl_ = observation; µ = population mean; α_i_ = effect of diet i (i = NC, PC, and GE); β_j_ = effect of sex j (j = male or female); δ_k_ = effect of sampling day k (k = (0, 6), 27/28, and 55/56); (αβ)ij = interaction between diet and sex; (αδ)_ik_ = interaction between diet and sampling day; (βδ)_jk_ = interaction between sex and sampling day; (αβδ)_ijk_ = interaction between diet, sex and sampling day; and ε_ijkl_ = residual error.

The 2∆∆Ct method was used to determine gene expression. First, the ∆Ct (normalized Ct) value for each sample was calculated by subtracting the Ct value for the target gene from the mean Ct value of the two reference genes. For each gene, the mean ∆Ct for each experimental group was calculated and subsequently used for statistical evaluation and expressing the fold change (=2∆∆Ct value). Fold changes higher than 1.5 and lower than 0.75 were considered to be physiologically relevant.

The least square means (LSMeans) were compared with the Tukey–Kramer test, and differences were considered statistically significant at *p* < 0.05 or as trends for *p*-values of between 0.05 and 0.10. The values of diets and sex in the tables are presented as LSMeans with pooled standard errors of means (SEM). For each parameter, tables display the means of the main effects of diet and sex in the first row, whereas the results of interactions between diet and sampling day, as well as the interaction between sex and sampling day, are presented in rows: rows two and three for the tissue analyses and rows two to four/five for blood analyses. However, post hoc tests in interactions were only performed for diets within one sampling day, not for different sampling days within one diet. The means of the main effects depict calculated mean values comprising all sampling days/both sexes. The means of the main effect of the sampling day are not shown in the tables. In the case of significant interactions (diet × sex; diet × d), no post hoc tests for the main effects of diet and sex were performed. Details for the interaction between diet and sex are shown in Supplementary Tables.

## 3. Results

### 3.1. Intestinal Morphology

The results of morphological analyses from the small intestine (jejunum and ileum) and colon are presented in Table 2 and Table 3, with additional material in Supplementary Tables S1 and S2.

Alterations in villus morphology were observed at both sampling points and for both small intestinal sections. Due to GE supplementation, increases in villus height (compared to NC and PC) and villus surface area (compared to NC) were observed in the jejunum on day 27/28. However, on day 55/56 in both the GE and PC groups, we found increased villus height and villus surface area in the ileum. This effect in the ileum was accompanied by a higher VC ratio and number of goblet cells in GE and PC for this sampling point. Furthermore, due to similar effects on the jejunal VC ratio for both sampling points, a higher VC ratio was detected for the main effect of the diet in GE compared to PC. The main effect of sex had no impact on villus measurements in the tissue of the small intestine. However, male piglets from the GE group showed an overall increased jejunal villus height (compared to the NC and PC) and increased villus surface area (compared to the NC; Supplementary Tables S1 and S2). Regarding the effect of the sampling day, most of the villus parameters analysed in the small intestine increased as expected on day 55/56 compared to day 27/28 (*p* < 0.05), with the exception of villus goblet cells in the jejunum and the VC ratio in the ileum.

The crypt morphology of the small intestine was not affected by any diet in either of the sampling points. However, in the colon, PC led to a lower crypt surface area compared to the NC (main effect of diet). Regarding an effect of sex, female animals demonstrated increased crypt depths and numbers of goblet cells from the jejunum, predominantly on day 27/28. There was no effect of sex in the tissue from the ileum and colon. Regarding the effect of the sampling day, crypt depth increased in the jejunum on day 55/56 in comparison to that on day 27/28. In the ileum, an increase in all crypt measurements was observed at day 55/56 compared to day 27/28 (*p* < 0.05). However, there was no effect of the sampling day on colon crypt morphology as the piglet aged.

The thickness of tunica muscularis increased with the age of the piglets (*p* < 0.05) in all three parts of the intestine. However, diets and sex showed no effects in either the small intestine or the colon (Supplementary Materials, Table S3).

### 3.2. Tissue Antioxidant Status Capacity and Gene Analyses

#### 3.2.1. Antioxidant Enzyme Activity, TBARS, DPPH and ABTS

Antioxidant status in relation to grape extract supplementation was evaluated from the intestinal tissue (jejunum and ileum) and liver. Results regarding antioxidant enzyme activity, as well as TBARS, DPPH, and ABTS, are shown in Table 4, Table 5 and Table 6.

The antioxidant enzyme activity of GPx was not affected by diet at either sampling point in the jejunum, ileum, or liver. However, a general increase in enzyme activity (*p* < 0.05) on day 55/56 compared to day 27/28 was observed in all three sampled parts of the intestinal tract.

For total SOD and Mn-SOD activity, there was no effect of the diets in the jejunum, ileum and liver. We observed an effect of the sampling day, increased activity on d 55/56 (*p* < 0.05) in the liver but not in the jejunum and ileum. The activity of Cu/Zn-SOD was not affected by the diets in the ileum and liver at any sampling point. However, in the jejunum, the PC tended to decrease enzyme activity (*p* < 0.10) compared to the NC on day 55/56. Regarding the sampling day, there were changes in the enzyme activity on day 55/56 in the tissue from the ileum (increase) and liver (decrease) but not from the jejunum.

The activity of CAT was not affected by the diets in the jejunum and liver, but we observed a tendency (*p* < 0.10) in the ileum of the PC to increase enzyme activity compared to the NC on day 27/28. Sampling day affected CAT activity (*p* < 0.05) in the ileum (decrease) and liver (increase) on day 55/56 compared to day 27/28.

Even though TBARS concentrations were not affected by the diets in the jejunum, ileum, and liver, an effect of the sampling day was observed in the jejunum. Here, TBARS concentrations decreased on day 55/56 (*p* < 0.05) compared to day 27/28. Additionally, an effect of sex was observed in the ileum on day 55/56, as male piglets showed higher TBARS concentrations compared to females.

For antioxidant capacity measured by DPPH and ABTS, we observed no differences between the diets in the jejunum, ileum and liver. In general, the values of DPPH and ABTS in the jejunum and liver increased on day 55/56 (*p* < 0.05). In the ileum, there was no effect of the sampling day; however, we observed an effect of sex for ABTS on day 55/56, with higher values in male animals. Concerning the interaction of diet and sex, we observed some effect on day 55/56, where male piglets from NC increased DPPH values from the ileum compared to the female piglets from the same treatment group (*p* = 0.011; means not shown). In addition, female piglets from the PC increased the ABTS values from the jejunum compared to the NC (*p* < 0.001; means not shown) on day 55/56. Additionally, within the PC, females increased ABTS values from the jejunum compared to males (*p* = 0.002; means not shown).

#### 3.2.2. Gene Expression

The effects of the grape extract supplementation on the expression of selected genes were evaluated in the jejunum, ileum and liver on two different sampling days (day 27/28 and day 55/56). These results are shown in Table 7, Table 8 and Table 9.

Genes involved in oxidative stress response were selected for the analyses in the liver, jejunum and ileum based on the selection of antioxidant enzyme activity from the tissue in the present trial. These included GPX2 (jejunum and ileum), GPX1 (liver), and SOD1 and CAT in the jejunum, ileum and liver. The expression of these genes was not affected by any diet at either sampling point. In the jejunum, an effect of the sampling day was observed for GPX2 and SOD1 expression (lower expression on day 55/56 than on day 27/28) but not for CAT, whereas in the ileum, we observed this effect for CAT expression but not for GPX2 and SOD1. In the liver, we observed increased expression on day 27/28 compared to day 55/56 (*p* < 0.05) for all three antioxidant genes (GPX1, SOD1, and CAT). There was no effect of sex on the expression of these genes.

In the tissue of the jejunum and ileum, we additionally analysed selected genes involved in tissue remodelling (ADAM8, COL4A4, COL7A1, and LAMB3) and one immune-response-related gene (SPP1). We observed no effect of the diets in the jejunum. On d 55/56 in the ileum, the expression of ADAM8 abundance was upregulated in the PC group compared to the GE group. The expression of COL4A4 increased on day 55/56 (*p* < 0.05) in both the jejunum and ileum. Additionally, this effect was observed in the jejunum for SPP1 expression. Apart from the downregulation of LAMB3 in the ileal tissue of female animals on day 27/28, no additional effects of sex were observed for these genes.

In the liver, we additionally investigated the relation of GE supplementation to the expression of selected stress and tissue-repair-related genes (heat shock proteins HSP70 and HSP90AA1, CYP8b1, and MMP-13) and genes involved in inflammation (TNFRSF14 and CCL4). There were no changes in the expression of these genes related to any diet in individual sampling days. Though significant, the upregulation of GE compared to the NC for the stress marker HSP70 (main effect of diet) was still below the physiological relevance level of 1.5. We observed an increased expression of CCL4 on day 55/56 compared to day 27/28. We also analysed the expression of sterol 12α-hydroxylase, cytochrome P450 8B1 (CYP8b1), a gene involved in bile acid homeostasis, in the liver. On day 27/28, CYP8B1 was downregulated to 0.24-fold (200% decrease) in the liver tissue of piglets from the PC, whereas the GE group showed an almost identical expression as the NC. Matrix metalloproteinase 13 precursor (MMP-13) is a gene related to processes in tissue repair during inflammatory processes [49]. The expression of MMP-13 was not shown to be influenced by any dietary treatment on individual sampling days. There was no effect of sex on the expression of these genes. However, CYP8B1 was overall upregulated by male piglets from the PC compared to the NC and GE. Moreover, also within the PC group, CYP8B1 expression was significantly higher in male than female animals (diet × sex; *p* < 0.05; Supplementary Materials, Table S4).

### 3.3. Plasma Antioxidant Measurements and Acute-Phase Proteins (APPs)

The results of plasma antioxidant measurements, as well as those for APPs (haptoglobin and PigMAP (major acute-phase protein)), are shown in Table 10.

On day 6, piglets from the GE group had lower MDA concentrations compared to the PC but no difference from the NC. Dietary treatments did not affect plasma SOD and acute-phase proteins on any sampling day. We observed an effect of sex on day 55/56, where female animals showed higher haptoglobin concentrations.

**Figure 2:**
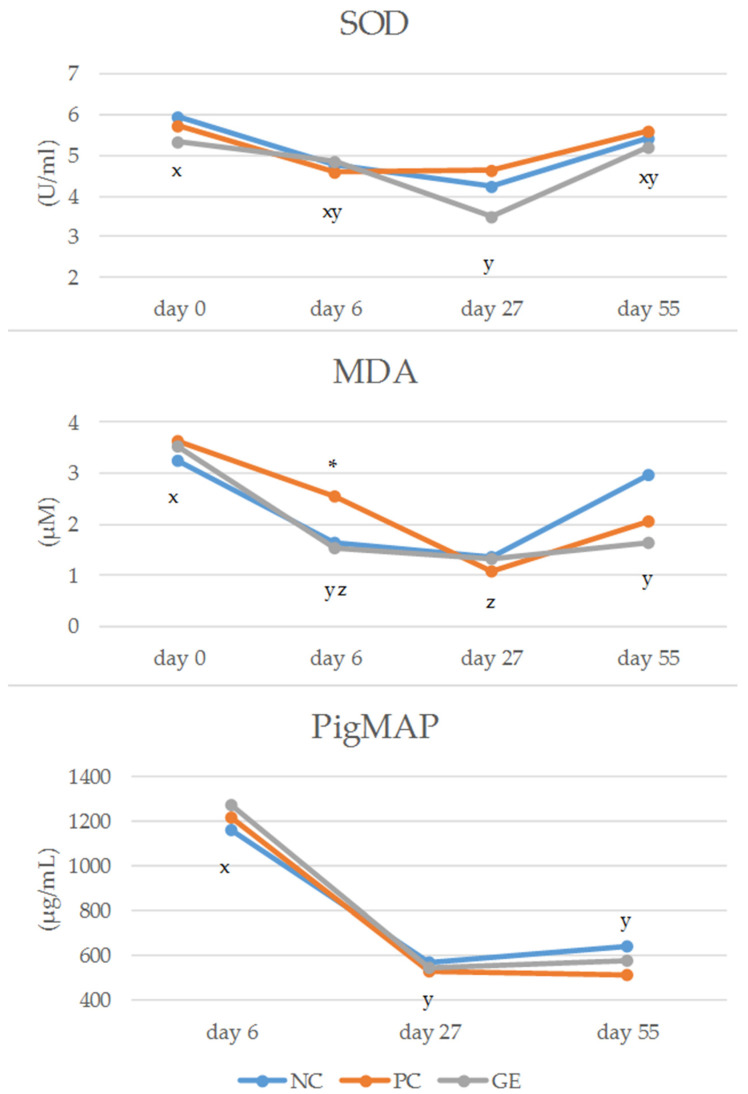
Main effect of sampling day (d) on plasma antioxidant measurements (SOD, superoxide dismutase; MDA, malondialdehyde) and acute-phase proteins (PigMAP (major acute-phase protein). LSMeans of the respective diets are shown for every sampling day. x, y, z: means of the fixed effect sampling day (d) with different letters significantly differ (*p* < 0.05). * Effect of the diet observed; further information in Table 10.

A major effect regarding plasma analyses was observed for the main effect of sampling day, with the exception of haptoglobin (Figure 2). Plasma SOD decreased in the first part of the trial and thus differed on day 27/28 compared to the values on day 0 (*p* = 0.017). On day 55/56, plasma SOD again increased, though without differing from the values on any other sampling day. Plasma MDA levels decreased compared to the blood sampling on day 0, with lowest values on day 27/28. Plasma pigMAP evidently decreased on day 27/28 and day 55/56 compared to the concentrations measured on day 6 (*p* < 0.001). For all measurements from plasma, an effect of sex was observed only for haptoglobin on day 55/56, with higher values in female animals compared to male animals.

## 4. Discussion

The challenges of high disease incidence and economic losses in intensive pig husbandry often accompany the critical weaning phase of piglets. Circumstances during weaning, where piglets are often exposed to an increased overgrowth of pathogens such as enterotoxigenic *E. coli* (ETEC) and *Salmonella* [50,51], include antibiotic growth promoter (AGP) supplementation (if permitted) or require the antibiotic treatment of gastrointestinal symptoms [52]. Piglets from the present trial also showed diarrhoea symptoms post weaning that persisted for the first 3 weeks of the trial. Analysed faecal samples from different treatment groups were tested positive, inter alia, on ETEC strain K88 [38]. By reducing the intestinal overload of pathogens, antibiotics reduce the diarrhoea incidence at weaning and improve piglets’ performance [22].

Antibiotics are powerful medicines; however, their improper use may also accelerate the increasing threat of antibiotic resistance, with negative consequences for the environment, animal health, and human health [23]. Many research groups have explored the potential of different plant-derived feed additives (including grape polyphenols) on the diarrhoea, nutrient digestibility and performance of the piglets [53,54,55,56], aiming for alternatives to AGP or antibiotic treatments. It is important to understand the mechanisms behind a secondary plant metabolite diet substitution if the aim is to replace or at least reduce routine preventive antibiotic usage at weaning. The potential of grape polyphenols in the gut (antimicrobial, antioxidative, and immunomodulatory) is an important opportunity in this direction [29,57,58,59]. However, besides economic factors, there has been inconsistency in the results of using such plant alternatives until now. Different trial conditions, synergies, and antagonisms between different compounds in the diets, as well as a common lack of proper characterization of used compounds, are among the circumstances that led to said inconsistencies. For the present trial, applied GE was specified with ~40% total polyphenol content and added with 150 g/t of the diet according to supplier’s recommendations. Accordingly, a grape-derived polyphenol supplementation level of 6 mg gallic acid equivalents (GAE)/100 g FM was calculated [38]. For the present publication, we evaluated the potential of GE on the gut and liver tissue levels, as well on the systemic level in the blood, with the aim to gain more insight on how GE might improve nutrient digestibility. Additionally, it is important to note that we evaluated the effect of GE inclusion in comparison to a NC and the therapeutic antibiotic treatment as the PC, which had one aggravating circumstance regarding result interpretation. To our knowledge, most previous studies with pigs using grape polyphenols and a PC in the study design were performed with antibiotic growth promoters (subtherapeutic antibiotics in feed for the whole trial duration) as the PC [26,27]. This is an important difference to the dosage and duration of the antibiotic treatment in the present work and therefore limits direct comparison between results.

### 4.1. Intestinal Morphology

The intestinal epithelium is a single layer of intestinal epithelial cells organized into villi and crypts [9]. Such a complex organization makes the intestinal epithelium a critical component of the intestinal barrier and explains the close relation between the degree of maturation and absorptive function of the intestine [60]. The barrier disruption (e.g., at weaning) provokes infection, inflammation, and changes in intestinal morphology, such as villus atrophy and crypt hypertrophy, as a reaction to the activation of immune response due to digestive disorders caused by, e.g., pathogens [61,62]. Insight into the intestinal morphology therefore provides valuable information regarding the absorptive function of the intestine.

Villus and crypt architecture: In the present work, we observed positive effects of diets on small intestinal villus measurements at both sampling points. The GE supplementation increased villus height (compared to the NC and PC) and villus surface area (compared to the NC) in the jejunum on day 27/28. In line with our observations, in a study by Han et al. [26] with weaning piglets (28 days trial duration), jejunum villus height was greater in the grape seed pomace group than that of a negative control. However, they also showed positive villus height development in an AGP group. In the present work, ileum villus height was not increased on day 27/28, as was the case in the study of Han et al. [26]. However, our results showed an increase in villus height, villus surface area, and villus goblet cells from the ileum on day 55/56 by GE and PC compared to the NC. An enhanced density of goblet cells in the small intestine due to grape seed extract was reported in a study with mice [63]. An increased number of mucus-producing and-secreting goblet cells may suggest an increased capacity for mucus production in the intestinal lumen. Mucus has a protective (against physicochemical damage and adherence of pathogens) and regulatory (in regard to passage of intestinal content) role within the gut [64].

Regarding the VC ratio measurements from the present study, we observed similar developments in the jejunum at both sampling points (day 27/28 and day 55/56), which led to an increased VC ratio of the main effect of diet in the GE group compared to the PC (*p* < 0.05) but not compared to the NC. In the ileum, GE and PC increased the VC ratio compared to the NC on day 55/56. A higher VC ratio may present stronger abilities for epithelium turnover and nutrient absorption, as previously reported for grape polyphenols and AGP [65,66]. Regarding the sampling days and effects on villus morphology in two different parts of the small intestine, unlike in the jejunum, the ileum effects were more pronounced on day 55/56 than day 27/28. Another interesting observation was seen regarding the effect of the PC, with no effect in the jejunum and an effect in the ileum comparable to those of the GE group. We suppose that the ileum, which generally has a longer recovery time [67] and a higher number of goblet cells [2] compared to the jejunum, needed more time post weaning to reach the maturation condition that enabled positive effects of dietary interventions (PC and GE in the present work).

Our results additionally showed an interaction between the main effects of diet and sex in the jejunum as an increase in the villus height (compared to the NC and PC) and a tendency to increase villus surface area on day 55/56 by the male animals from the GE group (diet × sex; *p* < 0.05). This slight effect explains the numerical advantage of GE (*p* > 0.10) to the PC and NC at the end of the trial (day 55/56), though without a significant effect on villus measurements. Similarly, male young chicks in a previous study showed increased jejunum villi length compared to females [68].

The morphological evaluation of crypt architecture from the jejunum, ileum and colon indicated no effect of the diets at respective sampling points. This is in contrast to the findings from Han et al. [26], where grape seed and AGP reduced the crypt depth in weaning piglets after 28 days. Schwarz et al. [69] tested different concentrations of grape extract in a broiler diet on different crypt measurements and found a quadratic effect on the crypt depth (an increase) with increased grape extract concentration. However, regarding the main effect of diet in the present work, as a result of the similar effects at both sampling points, the PC showed significantly lower crypt surface area compared to the NC. Smaller crypts, in general, reflect lower tissue turnover and a lower demand for villus renewal and tissue synthesis [2], which indicates overall lower pathogen overload in PC. However, there was no clear effect of any diet at either sampling point, which allows us to infer that the likelihood of infection and the occurrence of pathogens was probably similar, with an overall slight advantage of the PC at some point. An effect of sex was observed only in the jejunum, where female animals showed increased crypt depth and number of goblet cells on day 27/28. Similar results for crypt depth were found in young female chicks compared to male chicks, as reported by Gimenez et al. [68]. In this study, the effect of the sampling day (on day 55/56 compared to day 27/28) was more pronounced in the ileum (all crypt measurements increased) than in the jejunum (increased crypt depth).

Tunica muscularis thickness: We measured the thickness of tunica muscularis mucosae with regard to the role of the muscularis layers for gradually moving the luminal contents along the gastrointestinal tract. Adapting to a new type of feed at weaning also affects gut motility [2]. Peristaltic movement is responsible for the blending of the gut content and increases enzymatic activity on nutrients, thereby affecting the absorption of nutrients [70]. A diet-induced thickening of the muscularis layer was observed in a broiler study with grape seed extract after 21 days [71]. However, after an additional 14 days of the trial, where the birds were exposed to heat stress, the effect of grape seed extract on the muscularis thickness was no longer observed. Viveros et al. [66] observed an increase in jejunal muscularis thickness in birds fed grape seed extract and grape pomace concentrate, but also AGP, when compared to a negative control. In the present work, there was no impact of the diet and sex on the tunica muscularis thickness in the small intestine and colon.

Higher villi, increased villus surface area, and increased VC ratio are indicators of the absorption and digestion capability of intestinal villi [2]. Villus hyperplasia, as achieved by GE and partly by the PC in this study, may be regarded as favourable, as it indicates an absence of undesirable infection features such as villus atrophy [1]. Moreover, an increased number of goblet cells, as observed in the present study (ileum on day 55/56), may suggest accelerated cell turnover with reduced potential for bacterial adhesion in the intestinal epithelium, thus beneficially affecting nutrient digestion and absorption processes [72]. Even if no clear effect on crypt architecture was observed in respective sampling points, an advantage of the PC compared to the NC for crypt area in regard to the main effect of diet may additionally indicate a lower occurrence of pathogens and infections, which seems to be comparable between GE and PC for both sampling points. Together, these observations indicate that GE may have a role in protecting intestinal mucosa and functional integrity at the same level or partly over the level of the PC. These digestion- and absorption-favouring circumstances from intestinal morphology allow us to link these results to the results from our previous work [38], where the improvement of nutrient digestibility (partly over the level of PC) was achieved following GE supplementation.

### 4.2. Tissue and Plasma Antioxidant Capacity

Polyphenols can act directly as antioxidants owing to electron-donating phenolic groups in their structures [73]. Another mode of action is more indirect: the activation of specific stress-sensitive transcription factors, which increase the level of antioxidant enzymes, different cytoprotective genes, and genes involved in anti-inflammatory processes, thus reducing the noxious effects of ROS [74].

#### 4.2.1. Antioxidant Enzyme Activity of Jejunum, Ileum and Liver Tissue

Antioxidant enzymes (e.g., GPx, SOD, and CAT) are crucial components in ROS reduction [75]. Taranu et al. [76] demonstrated a decrease in antioxidant enzyme activity in the duodenum, colon and liver of weaning piglets fed with an aflatoxin-loaded diet compared to those fed grape by-products or a negative control. Moreover, grape seed meal inclusion in the diet containing aflatoxins was able to restore antioxidant enzyme activity and counteract oxidative damage induced by mycotoxins. In the liver, antioxidant capacity was clearly enhanced compared to a negative control. In the present work, there was no effect of the diets on either sampling day on GPx, total SOD, Mn-SOD or Cu/Zn-SOD activity in the ileum and liver. For GPx, this was in agreement with observations of Chedea et al. [31], who detected no effects of grape supplementations on GPx activity in liver of piglets fed grape pomace. However, in the same study, an increased SOD activity in the liver was found. We observed a tendency of the PC to decrease Cu/Zn-SOD activity (*p* < 0.10) compared to the NC on day 55/56 in the jejunum. Concerning the effect of sampling, we measured an increase in GPx activity on day 55/56 compared to day 27/28 in all three sampled parts of the intestine. With the exception of the jejunum, SOD activity remarkably changed (*p* < 0.05) on day 55/56 compared to day 27/28: in the liver, the total SOD and Mg-SOD increased while Cu/Zn-SOD decreased, and in the ileum, Cu/Zn-SOD also increased.

In measuring the activity of CAT, we observed a tendency (*p* < 0.10) of the PC to increase enzyme activity compared to the NC on day 27/28 in the ileum. There was no effect of diets in the jejunum and liver. Chedea et al. [31] reported a tendency for increasing CAT activity in the liver of piglets fed grape pomace; however, in our study, GE supplementation did not affect CAT activity at either sampling day. In our study, CAT activity changed (*p* < 0.05) in the ileum (decrease) and liver (increase) on day 55/56 compared to day 27/28.

The induction of antioxidant enzyme activity increases cell protection and the elimination of ROS. On the other hand, some authors have speculated that the presence of exogenous (e.g., dietary) antioxidants in the systemic circulation may even reduce the need for higher antioxidant enzyme activity in the organs or serum [77]. The present study does not provide information on the bioavailability of GE polyphenols to discuss its contribution to the antioxidant environment. Our findings, however, did not suggest any advantage of GE in relation to the PC or NC regarding antioxidant enzyme activity for the selected sampling points.

#### 4.2.2. Antioxidant Capacity of Jejunum, Ileum and Liver Tissue Measured by DPPH, ABTS and TBARS Assays

Antioxidant capacity was additionally determined by the DPPH and ABTH methods in the jejunum, ileum and liver, but it remained unchanged at both sampling points. These are two commonly used methods to estimate the ability of different dietary compounds to reduce ROS by observing the absorbance changes of the stable and coloured radicals DPPH and ABTS [78]. The TBARS assay is used to determine the lipid oxidation level by measuring (most commonly) the reaction of malondialdehyde (MDA) with thiobarbituric acid (TBA), a by-product of lipid peroxidation [79]. The concentration of TBARS can be elevated in different diseases and increased oxidative stress by noxious agents, e.g., pathogens or toxins [76]. Previous studies with piglets fed grape pomace [31,57] also reported lower lipid oxidation in several tissues (including the liver) of piglets fed grape pomace compared to a negative control. However, our current results showed no effect of the diets on TBARS concentrations in the jejunum, ileum or liver of the piglets. This is consistent with findings regarding the TBARS level in the liver made by Gessner et al. [80] in a study with piglets fed grape seed and grape marc extract. A possible explanation for these discrepancies might be the level of oxidative stress (regarding the sampling day and weaning stressors) or the inclusion level of grape polyphenols.

We also observed an effect of sex on these measurements in the ileum on day 55/56, where ABTS and TBARS values were increased in male piglets. Research on the effect of the animal’s sex on antioxidant capacity measured by ABTS in pigs is, to our knowledge, limited. We previously mentioned the role of mast cell proinflammatory mediators (e.g., cytokines, proteases, and histamines) in the alleviation of oxidative stress level. They induce ROS release by cells [12]. It may be assumed that the sex-related increase in the production of proinflammatory mediators also affects the antioxidant response of cells (e.g., in ABTS measurements). Pohl et al. [81] reported higher release in mass cell numbers and diarrhoea incidence in female piglets exposed to weaning stress compared to male piglets. That study allowed us to expect a higher tissue antioxidant capacity (e.g., increase in ABTS measurements) in male animals, which could be an explanation for our results seen in the ileum. However, it is important to mention that in the present study, no correlation between diarrhoea incidence and mast cell release was examined. A possible explanation for higher TBARS values in male animals might be the correlation of fat accumulation with oxidative stress markers, e.g., TBARS accumulation [82]. Furthermore, at the same sampling point, DPPH values in the ileum increased in male piglets from the NC compared to the females (diet × sex; *p* < 0.05). Interestingly, in the jejunum, female animals from the PC increased ABTS measurements compared to female animals from the NC, and within the PC, females had higher ABTS values compared to males (diet × sex; *p* < 0.05).

The PC, which was applied in the first five days post weaning, also showed no effect on antioxidant measurements from the liver, with minor effects in the jejunum and ileum compared to the NC. In general, the effect of antibiotic on antioxidant measurements is expected to be an indirect one that occurs through the modulation of the microbial load. Additionally, amoxicillin, as used in the present trial in the PC group, may even have a certain hepatotoxicity [83]. However, the results from the present study did not show any changes in the measurements from the liver that would indicate increased oxidative stress due to the hepatotoxicity of antibiotic treatment. It can be questioned if the time of tissue sampling was set too late post weaning, in terms of the continuously evolving antioxidant features, especially in regard to a certain challenge of the piglets due to the presence of *E. coli* at the beginning of the present trial.

#### 4.2.3. Plasma Antioxidant Measurements

Depending on the structural diversity and polyphenol subfamily, as well as the intestinal microbiome and enzyme activity, biotransformations of dietary polyphenols affect antioxidant indices are measured in serum [84]. For a better insight in the antioxidant response, we determined SOD and MDA levels in the plasma of weaning piglets.

Regarding SOD and MDA measurements, the sampling days 0 (two days before weaning) and 6 (after antibiotic treatment in the PC group) should highlight how these measurements might have been elevated by the effect of weaning. In particular, it was considered of interest to see whether there was an effect of the initial antibiotic treatment and whether it was comparable to the potential of GE in this phase of the piglet’s life, and we did observe a strong effect of the sampling day for SOD and MDA measurements (Figure 1). Considering the effect of the weaning, only MDA measurements decreased compared to those taken before weaning (day 0), although the animals from this trial were found positive for *E. coli* and a certain effect was expected in terms potential cell damage and SOD increase provoked by the *E. coli* infection [55]. There were contrary findings reported in a study by Stukelj et al. [85], with viral-infection-induced oxidative stress and higher SOD activity in weaners and fatteners but not in finisher pigs compared to the corresponding negative control. In the present work, we did not observe any effect of the treatment groups on SOD measurement in plasma on any of the four sampling days. In contrast, in the study of Hao et al. [27] where different supplementation levels of grape seed proanthocyanidins were tested in relation to AGP and a negative control in the diet of weaning piglets, the highest grape seed dosage increased serum SOD levels on day 14 and day 28 compared to a negative control. Furthermore, grape seed supplementation increased SOD level in comparison to AGP, but only on day 28. In another study, grape seed proanthocyanidins diet supplementation increased serum SOD activity [86] compared to the negative control, though without significant difference to AGP-fed piglets. In studies with broilers [87] grape seed supplementation was also able to enhance SOD activity. The animals from our trial generally showed the highest SOD concentration on day 0. Those concentrations significantly differed from the values on day 27/28, which were the lowest of all measurements. Similar results regarding the effect of the sampling day were observed for MDA concentration. As mentioned above, MDA is an indicator of lipid peroxidation, which may increase following cellular damage due to increased ROS formation [87]. Grape seed extracts were tested in two different concentrations in the diet of Pekin ducks [65] and found to linearly decrease serum MDA. Surprisingly, in the present work, the MDA level tended to increase (*p* < 0.10) after 5 days antibiotic treatment in the PC group compared to the GE group, thus suggesting potential cellular damage induced by antibiotic treatment. However, even if not completely comparable to the antibiotic treatment in our trial, Hao et al. [27] also observed an increase in the MDA level by their AGP diet supplementation compared to three different grape seed proanthocyanidin supplementation levels (on day 14 and day 28), as well as when compared to negative control (only on day 14). They also showed that the three levels of grape seed supplementation exerted different effects, which grew more pronounced with increasing polyphenol supplementation, although the level of grape polyphenols in the product used in that study was not specified. The highest values for SOD and MDA at the beginning of the trial allows us to assume that there was a certain effect of weaning regarding the level of (anti)oxidative activity at the beginning of the trial. Nevertheless, the GE and PC were not able to improve plasma oxidant status at this or some later point of the trial.

Our results from the tissue and plasma showed that imbalances in ROS production and elimination, which may be followed by the disruption of intestinal integrity, cannot be assumed. These results also do not seem to be a mechanism to explain the impact of GE on intestinal morphology and cannot be connected to the positive effects in nutrient digestibility from our previous study [38]. The absence of effects from the grape extract in the present study may have been a consequence of the moderate grape extract supplementation level in the diet, although this was selected according to the manufacturer’s recommendation.

### 4.3. Gene Expression Analyses from Jejunum, Ileum and Liver Tissue

Cells of different organs that fail to maintain redox balance may be affected in their functionality [88]. In general, an organism reacts promptly and in different ways to prevent the disruption of cell turnover and apoptosis, DNA and protein damage, and eventual organ dysfunctionality [18]. With this in mind, we investigated some marker genes of oxidative stress and tissue remodelling (small intestine). In the liver, we focused on marker genes of oxidative stress, as well as immune and stress-signalling related genes.

A strong inflammatory reaction at the early stage of immune response induces pathways that are positively correlated to the expression of antioxidant enzymes [89]. The potential of polyphenols in the prevention of excessive ROS production is interesting with regard to increased antioxidant enzyme production and in the regulation of heat shock proteins (HSPs). Both are associated with cytoprotection, as shown by Yin et al. [90]. Antioxidant genes are upregulated in order to convert the superoxide anion radical to H_2_O_2_ (e.g., SOD1) or they are involved in modulating of glutathione homeostasis (e.g., GPX1, GPX2, and CAT) [91]. Gessner et al. [80] assumed that the significant downregulation of SOD1 gene in the duodenum of pigs fed grape seed and grape marc extract was probably caused by strong polyphenol properties (grape polyphenol supplementation level of 850 mg GAE/100 g) and the low activation of signal pathway for genes (Nrf2 is activated by either ROS or by pro-inflammatory cytokines). In the present work, the expression of the targeted antioxidative genes in the jejunum and ileum (GPX2, SOD1, and CAT) and liver (GPX1, SOD1, and CAT) was not elevated by the diets at either of the sampling points.

Heat shock proteins HSP70 and HSP90AA1 play important roles in cellular response to stress (environment, psychological stress, transportation, heat stress, hypoxia, and food deprivation) [92]. A study by Zhang et al. [93] demonstrated that the livers of pigs that experienced a four-hour transportation stress reacted with higher HSP70 expression, and these results correlated with histopathological and plasma indices of cellular damage from the liver. Moreover, another study showed reduced HSP70 expression in organs after longer period (6 h) of transportation stress, thus suggesting a certain adaptability to the expression of this gene [94]. The role of polyphenols in the presence of heat stress and their potential in stimulation of HSPs was explored in chickens by Yin et al. [90], who showed no clear direction of effects of tea-derived phenolic compounds on different HSPs in the presence of heat stress. The results from our analyses in the liver, without changes in the expression of HSP70 and HSP90AA1 by the diets, suggest an absence of acute stress on day 27/28 and day 55/56 that could allow polyphenols to alleviate the expression of these genes. In addition, Negrato et al. [95] reported a higher susceptibility of male fattening pigs to intensive housing conditions compared to female pigs, which responded with a higher expression of HSP70. In the present work, however, we did not observe an effect of the sex of weaning piglets on HPS expression.

Polyphenols may also enhance immune function by suppressing pro-inflammatory pathways in the small intestine [80,96]. Grape polyphenols used in a study by Gessner et al. [97] showed no impact on the expression of hepatic genes involved in inflammatory processes. These findings are in agreement with our results. In the present work, the expression of selected pro-inflammatory marker genes in the liver (TNFRSF14 and CCL4) and small intestine (SPP1) was not alleviated by the diets. Compared to the study by Gessner et al. [97], who explained these results by the fact that the piglets were not subjected to any stressful events, the piglets from the present study experienced different stressors (e.g., blood sampling) post weaning.

Guided by the possibility that a certain anti-inflammatory reaction at weaning might induce tissue repair mechanisms, we analysed additional genes in the jejunum and ileum that play a role in the maintenance and repair of the intestinal mucosa (ADAM8, COL4A4, COL7A1, and LAMB3) and liver (MMP-13 and CYP8B1). The expression of these genes was not clearly elevated by the diets in the present trial. This was not surprising in regard to our results from crypt morphology, which also showed no effect of the diets on these measurements (low demand for tissue turnover). In a study with mice [98], grape by-products inhibited the activity of cytochrome P450 2E1, which was discussed as a cytoprotective attribute of these products. In the present analyses, on day 27/28, the expression of CYP8B1 (cytochrome P450 8B1) was decreased in the PC group compared to the GE and NC groups; however, due to the less than two-fold change, these changes were not considered physiologically relevant.

The results of gene expression in the tissue of the jejunum, ileum and liver showed that the supplementation of GE in the chosen dietary concentration had no physiologically relevant effects on the expression of genes involved in tissue remodelling, antioxidative stress, and immune response evaluated in the present study. Additionally, a likely explanation of the lack of dietary impact on stress and immune response-related genes might be the selection of the sampling points, which may have been set too late in regard to the expected challenge and inflammation following stressful events at weaning.

### 4.4. Plasma Acute-Phase Proteins (APPs)

The plasma biomarker APPs, as an integral part of innate immune system in the detection of acute inflammation, respond to inflammation, physical and physiological stress, and tissue injury [99], which include events such as diarrhoea, as observed at the beginning of the present trial (data not shown) [38].

Haptoglobin and pigMAP belong to a group of APPs that react to stimuli (e.g., transportation, stress, infection, and inflammation) with an increase in the blood [100,101,102]. However, in our trial, neither GE nor the PC showed potential to affect APPs. The most interesting results were observed for pigMAP regarding the sampling day (measured on days 6, 27/28 and 55/56). On day 6, we measured the highest concentrations, and these values decreased (*p* < 0.05) until day 27/28 and were maintained in this range until the end of the trial (day 55/56). These results are not surprising, in perspective to the issues (including diarrhoea) at the time of weaning. In a study with Göttingen minipigs [100], older female animals had significantly higher haptoglobin levels than the males, while the opposite effect was observed for pigMAP. Additionally, in the present work, a pronounced effect of the sex was observed on day 55/56, where female animals had significant higher haptoglobin concentrations compared to the male animals. However, pigMAP concentrations were not affected by sex. Similar, Pomorska-Mól et al. [103] found no gender-related effect on pigMAP levels in pig serum. Apart of the difference in a breed and a kind of animals used in the trial, the differences in the results may partly reflect age-dependent changes in regard to the sexual maturation or castration of pigs and minipigs [100].

## 5. Conclusions

The results from the present study on the tissue and plasma antioxidant capacity of GE do not support our hypothesis: GE supplementation did not lead to improved antioxidant ability compared to the levels measured in the PC or NC groups. However, the few well-studied key antioxidant enzymes and antioxidant capacity measurements may not reveal the whole story about the antioxidant capacity of GE used in this work. Stress-related plasma measurements indicated weaning issues; however, neither the GE nor PC showed evidence of a potential to alleviate the stress response. At the later sampling points, stress-related measurements from plasma reached a stable plateau corresponding to the low antioxidant activity found for GE and PC. Therefore, we conclude that the inclusion of the selected grape polyphenol did not achieve a desirable level of antioxidant activity. However, GE was favourable for the growth of small intestinal villi. Therefore, results from this study taken together with those from our previous work lead us to associate positive developments in the villus architecture with improvements in nutrient digestibility. Nutritional interventions with grape-derived polyphenols are promising steps to improve intestinal health of weaning piglets, although the mechanisms and effects still vary. We further suggest that the characterization of the intestinal microbiome may help to improve our understanding of the results obtained in the present study and our previous work.

## Figures and Tables

**Table 1:** Reference and selected genes used for analyses of gene expression.

Gene Symbol	Official Full Name	Reference Sequence
Jejunum and ileum		
COL4A4	Collagen, type IV, alpha 4	XM_013984558
COL7A1	Collagen, type VII, alpha 1	XM_005669519
SPP1	Secreted phosphorprotein 1	NM_214023
ADAM8	ADAM metallopeptidase domain 8	XM_013994286
SOD1	Superoxide dismutase 1, soluble	NM_001190422
GAPDH	Glyceraldehyde-3-phosphate dehydrogenase	NM_001206359
ACTB	Actin, beta	XM_003124280
LAMB3	Laminin, beta 3	XM_013989670
GPX2	Glutathione peroxidase 2 (gastrointestinal)	NM_001115136
CAT	Catalase	NM_214301
B2M	Beta-2-microglobulin	NM_213978
Liver		
GPX1	Glutathione perioxidase 1	NM_214201
CAT	Catalase	NM_214301
SOD1	Superoxide dismutase 1, soluble	NM_001190422
MMP-13	Matrix metalloproteinase 13 precursor	XM_003129808
TNFSF14	Tumour necrosis factor ligand superfamily member 14-like	NM_001260482
HSP70	Heat shock protein	NM_001123127
CYP8B1	Cytochrome P-450 8B1	NM_214426
HSP90AA1	90 kDa heat shock protein	NM_213973
CCL4	Chemokine (C–C motif) ligand 4	NM_213779
GAPDH	Glyceraldehyde-3-phosphate dehydrogenase	NM_001206359
ACTB	Actin, beta	XM_003124280

**Table 2:** Effect of dietary GE supplementation on intestinal villus morphology and VC ratio of weaning piglets compared to NC and PC.

Item ^4^		Diet ^1^			Sex		SEM ^2^	*p*-Value ^3^					
		NC	PC	GE	m	f		Diet	Sex	d	Diet × Sex	Diet × d	Sex × d
Villus height (µm)													
Jejunum													
Main effects		273	270	311	282	288	9.3	0.005	0.60	<0.001	0.008 ^§^	0.63	0.88
IA	day 27/28	229 ^b^	237 ^b^	278 ^a^	246	250	11	0.010	0.75		0.98		
	day 55/56	318	304	344	318	326	15	0.17	0.67		0.002		
Ileum													
Main effects		354	404	405	391	385	14	0.012	0.698	0.002	0.055	0.020	0.78
IA	day 27/28	361	366	362	368	358	17	0.98	0.608		0.14		
	day 55/56	347 ^b^	443 ^a^	448 ^a^	212	199	21	0.002	0.943		0.321		
Villus surface area (mm^2^)													
Jejunum													
Main effects		31.8	32.9	37.5	34.0	34.1	1.5	0.023	0.98	<0.001	0.006 ^§^	0.71	0.94
IA	day 27/28	24.4 ^b^	27.1 ^ab^	31.7 ^a^	27.8	27.7	1.7	0.016	0.96		0.35		
	day 55/56	39.2	38.7	43.5	39.9	41.1	2.6	0.35	0.77		0.023		
Ileum													
Main effects		53.4	62.6	62.9	60.6	58.6	2.3	0.006	0.46	<0.001	0.13	0.003	0.81
IA	day 27/28	53.4	52.7	52.7	53.6	52.3	2.9	0.98	0.69		0.040 *		
	day 55/56	53.4 ^b^	72.4 ^a^	73.1 ^a^	67.6	65.0	3.5	<0.001	0.53		0.72		
Villus goblet cells (*n*/villus)													
Jejunum													
Main effects		14.9	14.5	15.3	14.4	15.4	0.8	0.78	0.24	0.41	0.78	0.15	0.34
IA	day 27/28	13.9	15.4	14.3	13.6	15.5	1.1	0.63	0.15		0.79		
	day 55/56	15.9	13.6	16.3	15.3	15.4	1.1	0.18	0.86		0.29		
Ileum													
Main effects		12.5	14.7	14.9	13.8	14.3	0.7	0.024	0.48	<0.001	0.72	0.035	0.27
IA	day 27/28	12.1	12.2	12.3	12.4	12.1	0.9	0.99	0.79		0.40		
	day 55/56	12.9 ^b^	17.1 ^a^	17.6 ^a^	15.2	16.6	0.9	0.002	0.21		0.87		
VC ratio													
Jejunum													
Main effects		0.59 ^ab^	0.56 ^b^	0.64 ^a^	0.60	0.59	0.02	0.007	0.58	<0.001	0.15	0.21	0.15
IA	day 27/28	0.51	0.52	0.59	0.56	0.52	0.003	0.10	0.25		1.00		
	day 55/56	0.66	0.61	0.67	0.64	0.65	0.03	0.31	0.72		0.079		
Ileum													
Main effects		1.19	1.32	1.30	1.29	1.24	0.06	0.31	0.48	0.24	0.25	0.016	0.25
IA	day 27/28	1.39	1.28	1.27	1.42	1.34	0.1	0.68	0.27		0.65		
	day 55/56	0.99 ^b^	1.36 ^a^	1.33 ^a^	1.21	1.34	0.07	0.002	0.70		0.21		

VC ratio = villus height/crypt depth ratio; ^1^ NC = negative control; PC = positive control, NC and 20 mg amoxicillin/kg BW twice a day for the first 5 days of the trial; GE = NC and grape extract, 150 g/t. ^2^ SEM: standard error of mean based on LSMeans. ^3^ d (sampling day): day 27/28 and day 55/56 = day of the trial/post weaning. ^4^ Main effects: main effects of diet and sex = mean values at day 27/28 and day 55/56; IA: interactions: diet × d and sex × d; ^a,b^ Values within a row without a common superscript significantly differ at *p* < 0.05. * Significant ANOVA with non-significant post hoc Tukey–Kramer test (*p* > 0.05). ^§^ Details of the interactions are shown in Supplementary Materials (Tables S1 and S2).

**Table 3:** Effect of dietary GE supplementation on intestinal crypt morphology of weaning piglets compared to NC and PC.

Item ^4^		Diet ^1^			Sex		SEM ^2^	*p*-Value ^3^					
		NC	PC	GE	m	f		Diet	Sex	d	Diet × Sex	Diet × d	Sex × d
Crypt depth (µm)													
Jejunum													
Main effects		478	490	498	480	499	7.3	0.16	0.027	<0.001	0.55	0.90	0.06
IA	day 27/28	461	469	478	451	487	13	0.67	0.024		0.91		
	day 55/56	495	512	519	507	510	13	0.42	0.84		0.67		
Ileum													
Main effects		323	321	325	319	327	11	0.98	0.56	<0.001	0.90	0.36	0.33
IA	day 27/28	283	302	299	285	305	16	0.65	0.29		0.60		
	day 55/56	364	340	350	354	349	15	0.54	0.77		0.65		
Colon													
Main effects		529 ^(a)^	492 ^(b)^	506 ^(ab)^	505	513	12	0.084	0.58	0.80	0.98	0.59	0.23
IA	day 27/28	527	497	496	494	520	19	0.41	0.23		0.29		
	day 55/56	528	487	517	515	506	15	0.13	0.60		0.16		
Crypt surface area (mm^2^)													
Jejunum													
Main effects		26.4	27.5	27.0	26.1	27.8	0.7	0.55	0.033	0.51	0.20	0.83	0.41
IA	day 27/28	26.3	26.9	26.8	25.4	27.9	1.1	0.93	0.058		0.58		
	day 55/56	26.5	28.0	27.2	26.7	27.8	1.1	0.61	0.40		0.47		
Ileum													
Main effects		18.8	19.2	19.1	19.0	16.4	0.8	0.96	0.92	<0.001	0.65	0.74	0.36
IA	day 27/28	15.7	16.7	16.8	15.9	16.9	1.0	0.69	0.40		0.61		
	day 55/56	22.0	21.7	21.3	22.1	21.3	1.3	0.94	0.62		0.62		
Colon													
Main effects		37.2 ^a^	32.7 ^b^	34.5 ^ab^	35.5	34.2	1.3	0.047	0.35	0.97	0.51	0.83	0.52
IA	day 27/28	37.0	33.3	34.4	35.1	34.8	1.7	0.31	0.94		0.83		
	day 55/56	37.4	32.1	35.0	36.2	33.7	1.8	0.15	0.29		0.11		
Crypt goblet cells (*n*/crypt)													
Jejunum													
Main effects		34.6	35.8	34.7	32.8	37.2	1.0	0.65	<0.001	0.71	0.83	0.57	0.54
IA	day 27/28	35.1	35.7	33.6	32.1	37.5	1.8	0.70	0.017		0.75		
	day 55/56	34.1	35.8	35.7	33.4	37.1	1.6	0.73	0.077		0.99		
Ileum													
Main effects		26.9	26.8	27.4	27.1	26.9	1.5	0.95	0.91	<0.001	0.58	0.23	1.00
IA	day 27/28	20.2	22.0	24.3	22.3	22.1	1.8	0.30	0.93		0.53		
	day 55/56	33.7	31.5	30.5	32.0	31.8	2.3	0.62	0.95		0.81		
Colon													
Main effects		52.8	50.1	51.7	52.6	50.5	1.5	0.45	0.22	0.059	0.14	0.092	0.90
IA	day 27/28	53.4	46.4	49.8	50.9	48.8	2.3	0.11	0.44		0.15		
	day 55/56	52.0	53.9	53.7	54.2	52.2	1.9	0.73	0.40		0.59		

^1^ NC = negative control; PC = positive control, NC and 20 mg amoxicillin/kg BW twice a day for the first 5 days of the trial; GE = NC and grape extract, 150 g/t; ^2^ SEM: Standard error of mean based on LSMeans; ^3^ d (sampling day): day 27/28 and day 55/56 = day of the trial/post weaning. ^4^ Main effects: main effects of diet and sex = mean values at day 27/28 and day 55/56; IA: interactions: diet × d and sex × d.; ^a,b^ Values within a row without a common superscript significantly differ at *p* < 0.05 (values within brackets *p* < 0.10).

**Table 4:** Effect of dietary GE supplementation on antioxidant enzyme (GPx and CAT) activity from the intestinal tissues and liver of the weaning piglets compared to NC and PC.

Item ^4^		Diet ^1^			Sex		SEM ^2^	*p*-Value ^3^					
		NC	PC	GE	m	f		Diet	Sex	d	Diet × Sex	Diet × d	Sex × d
GPx (U/g protein)													
Jejunum													
Main effects		169	179	175	176	173	8.6	0.75	0.81	0.033	0.27	0.50	0.36
IA	day 27/28	153	165	172	158	169	10	0.43	0.34		0.34		
	day 55/56	186	193	177	182	189	14	0.74	0.67		0.40		
Ileum													
Main effects		334	315	310	318	322	12	0.33	0.76	0.016	0.50	0.85	0.64
IA	day 27/28	312	298	298	304	302	20	0.83	0.92		0.98		
	day 55/56	356	332	323	332	342	14	0.23	0.51		0.083		
Liver													
Main effects		1119	1091	1078	1097	1095	40	0.76	0.95	<0.001	0.12	0.41	0.33
IA	day 27/28	987	1015	1018	985	1028	42	0.85	0.39		0.28		
	day 55/56	1251	1167	1139	1209	1161	67	0.48	0.54		0.12		
CAT (U/mg protein)													
Jejunum													
Main effects		71.6	71.5	72.5	71.1	72.6	2.5	0.95	0.61	0.19	0.89	0.99	0.72
IA	day 27/28	73.4	73.4	74.5	72.5	75.0	3.3	0.96	0.51		0.99		
	day 55/56	69.8	69.6	70.6	69.8	70.2	3.8	0.98	0.92		0.77		
Ileum													
Main effects		26.4	30.0	29.4	28.9	28.3	1.7	0.27	0.77	<0.001	0.65	0.06	0.68
IA	day 27/28	31.6 ^(b)^	40.3 ^(a)^	39.2 ^(ab)^	37.7	36.4	2.8	0.072	0.68		0.36		
	day 55/56	21.3	19.6	19.7	20.1	20.3	1.7	0.74	0.90		0.82		
Liver													
Main effects		8.5	9.5	8.6	9.0	8.9	0.6	0.47	0.88	<0.001	0.42	0.48	0.55
IA	day 27/28	7.0	8.8	6.8	7.3	7.7	1.0	0.31	0.77		0.61		
	day 55/56	10.1	10.3	10.5	10.6	10	0.8	0.96	0.55		0.68		

GPx = glutathione peroxidase; CAT = catalase; ^1^ NC = negative control; PC = positive control, NC and 20 mg amoxicillin/kg BW twice a day for the first 5 days of the trial; GE = NC and grape extract, 150 g/t; ^2^ SEM: Standard error of mean based on LSMeans; ^3^ d (sampling day): day 27/28 and day 55/56 = day of the trial/post weaning; ^4^ Main effects: main effects of diet and sex = mean values at day 27/28 and day 55/56; IA: interactions: diet × d and sex × d.; ^a,b^ Values within a row without a common superscript significantly differ at *p* < 0.05 (values within brackets *p* < 0.10);.

**Table 5:** Effect of dietary GE supplementation on superoxide dismutase activity from the intestinal tissue and liver of the weaning piglets compared to NC and PC.

Item ^4^		Diet ^1^			Sex		SEM ^2^	*p*-Value ^3^					
		NC	PC	GE	m	f		Diet	Sex	d	Diet × Sex	Diet × d	Sex × d
Total SOD (U/mg protein)													
Jejunum													
Main effects		37.9	31.4	34.7	35.9	33.4	2.5	0.19	0.38	0.62	0.95	0.38	0.46
IA	day 27/28	35.8	33.5	36.9	35.6	35.2	3.3	0.76	0.92		0.80		
	day 55/56	40.0	29.4	32.5	36.3	31.6	3.6	0.12	0.28		0.97		
Ileum													
Main effects		17.6	18.0	16.8	17.1	17.9	1.0	0.68	0.46	0.21	0.84	0.36	0.88
IA	day 27/28	17.8	16.2	16.3	16.4	17.1	0.9	0.40	0.53		0.82		
	day 55/56	17.5	19.7	17.3	17.7	18.7	1.7	0.53	0.61		0.61		
Liver													
Main effects		236	249	226	242	232	7.6	0.11	0.25	<0.001	0.89	0.68	0.74
IA	day 27/28	255	276	246	266	252	13	0.25	0.35		0.75		
	day 55/56	217	223	207	219	212	8.5	0.39	0.51		0.96		
Mn-SOD (U/mg protein)													
Jejunum													
Main effects		13.2	13.6	13.3	13.3	13.4	0.5	0.81	0.85	0.95	0.83	0.67	0.97
IA	day 27/28	12.9	14.0	13.1	13.3	13.4	0.7	0.52	0.86		0.46		
	day 55/56	13.4	13.3	13.4	13.3	13.4	0.8	0.98	0.92		0.95		
Ileum													
Main effects		9.70	9.37	9.02	9.62	9.21	0.4	0.50	0.45	0.95	0.15	0.37	0.75
IA	day 27/28	9.93	9.59	8.54	9.45	9.25	0.5	0.17	0.74		0.28		
	day 55/56	9.49	9.15	9.50	9.64	9.13	0.6	0.90	0.48		0.10		
Liver													
Main effects		35.8	39.1	35.8	37.3	36.4	1.8	0.35	0.65	<0.001	0.50	0.80	0.70
IA	day 27/28	40.3	45.3	41.3	42.3	42.2	3.2	0.51	0.97		0.22		
	day 55/56	31.3	32.8	30.2	32.3	30.6	1.7	0.58	0.38		0.52		
Cu/Zn-SOD (U/mg protein)													
Jejunum													
Main effects		24.7	17.8	21.5	22.7	20	2.3	0.11	0.32	0.58	0.97	0.36	0.42
IA	day 27/28	22.9	19.5	23.8	22.3	21.8	3.2	0.61	0.89		0.88		
	day 55/56	26.6 ^(a)^	16.1 ^(b)^	19.1 ^(ab)^	23	18.2	3.2	0.077	0.21		0.96		
Ileum													
Main effects		7.93	8.61	8.61	8.11	8.72	0.4	0.72	0.43	0.019	0.57	0.15	0.77
IA	day 27/28	7.86	6.63	7.78	7.02	7.74	0.9	0.53	0.39		0.87		
	day 55/56	8.00	10.6	9.40	9.14	9.54	1.1	0.25	0.76		0.48		
Liver													
Main effects		200	210	191	205	196	7.1	0.15	0.26	<0.001	0.72	0.80	0.58
IA	day 27/28	214	231	205	223	210	12	0.32	0.33		0.45		
	day 55/56	185	190	176	186	182	7.6	0.43	0.59		0.85		

SOD = superoxide dismutase; ^1^ NC = negative control; PC = positive control, NC and 20 mg amoxicillin/kg BW twice a day for the first 5 days of the trial; GE = NC and grape extract, 150 g/t; ^2^ SEM: Standard error of mean based on LSMeans. ^3^ d (sampling day): day 27/28 and day 55/56 = day of the trial/post weaning. ^4^ Main effects: main effects of diet and sex = mean values at day 27/28 and day 55/56; IA: interactions: diet × d and sex × d; ^a,b^ Values within a row without a common superscript significantly differ at *p* < 0.05 (values within brackets *p* < 0.10).

**Table 6:** Effect of dietary GE supplementation on lipid peroxidation (measured by thiobarbituric acid reactive substances (TBARS)) and antioxidant capacity (measured by 2,2-diphenyl-1-picrylhydrazyl (DPPH) and 2,2-azinobis (3-ethilenzotiazolin)-6sulfonate (ABTS)) from the intestinal tissue and liver of the weaning piglets compared to NC and PC.

Item ^4^		Diet ^1^			Sex		SEM ^2^	*p*-Value ^3^					
		NC	PC	GE	m	f		Diet	Sex	d	Diet × Sex	Diet × d	Sex × d
TBARS (μmol/kg) ^5^													
Jejunum													
Main effects		21.8	23.8	21.0	21.9	22.5	1.2	0.24	0.66	0.001	0.97	0.27	0.96
IA	day 27/28	23.4	27.9	22.9	24.4	25.1	2.0	0.16	0.77		0.54		
	day 55/56	20.3	19.7	19.1	19.4	20.0	1.4	0.84	0.73		0.43		
Ileum													
Main effects		14.6	15.7	16.1	15.5	15.4	0.9	0.45	0.94	0.15	0.94	0.28	0.02
IA	day 27/28	13.8	16.0	14.4	13.6	15.9	1.5	0.56	0.18		0.65		
	day 55/56	15.4	15.4	17.7	17.5	14.9	0.9	0.10	0.016		0.41		
Liver													
Main effects		70.2	70.2	72.9	68.4	73.8	2.9	0.75	0.11	0.58	0.82	0.43	0.82
IA	day 27/28	72.3	67.4	70.8	67.9	72.5	3.2	0.55	0.23		0.28		
	day 55/56	68.1	73.0	74.9	68.9	75.1	4.7	0.58	0.27		0.67		
DPPH (µmol/g) ^6^													
Jejunum													
Main effects		25.2	25.9	26.5	25.2	26.5	1.0	0.63	0.26	<0.001	0.33	0.94	0.99
IA	day 27/28	22.4	23.4	24.2	22.7	24.0	1.5	0.69	0.46		0.35		
	day 55/56	27.8	28.3	28.9	27.7	29.2	1.3	0.89	0.39		0.85		
Ileum													
Main effects		28.8	27.6	30.4	29.7	28.2	1.7	0.49	0.42	0.90	0.12	0.99	0.15
IA	day 27/28	28.9	27.8	30.6	28.5	29.7	2.4	0.72	0.66		0.61		
	day 55/56	28.7	27.5	30.3	31.0	26.7	2.3	0.68	0.10		0.011		
Liver													
Main effects		75.3	76.0	75.4	74.1	77.0	2.4	0.98	0.29	0.001	0.16	0.58	0.93
IA	day 27/28	70.4	73.3	69.2	69.3	72.5	3.5	0.70	0.44		0.70		
	day 55/56	80.2	78.7	81.6	78.8	81.5	3.3	0.82	0.48		0.14		
ABTS (μmol/g) ^6^													
Jejunum													
Main effects		33.3	34.6	34.7	34.0	34.4	0.6	0.21	0.65	<0.001	0.34	0.93	0.76
IA	day 27/28	31.3	32.3	32.8	32.1	32.2	1.1	0.60	0.93		0.36		
	day 55/56	35.4	36.8	36.7	36.0	36.6	0.6	0.15	0.42		<0.001		
Ileum													
Main effects		31.4	31.2	31.8	31.7	31.1	0.5	0.70	0.31	0.35	0.32	0.78	0.02
IA	day 27/28	31.1	31.2	31.3	30.8	31.5	0.8	0.98	0.44		0.81		
	day 55/56	31.7	31.2	32.3	32.7	30.7	0.6	0.43	0.007		0.21		
Liver													
Main effects		71.0	73.6	73.5	72.8	72.6	1.1	0.19	0.89	0.002	0.52	0.67	0.72
IA	day 27/28	68.4	72.3	71.0	70.4	70.7	1.7	0.25	0.89		0.38		
	day 55/56	73.6	75.0	76.0	75.2	74.5	1.6	0.57	0.72		0.80		

^1^ NC = negative control; PC = positive control, NC and 20 mg amoxicillin/kg BW twice a day for the first 5 days of the trial; GE = NC and grape extract, 150 g/t. ^2^ SEM: Standard error of mean based on LSMeans. ^3^ d (sampling day): day 27/28 and day 55/56 = day of the trial/post weaning. ^4^ Main effects: main effects of diet and sex = mean values at day 27/28 and day 55/56; IA: interactions: diet × d and sex × d; ^5^ Values expressed as µmol malondialdehyde equivalents per kg of wet tissue. ^6^ Trolox equivalents per g of wet tissue.

**Table 7:** Expression of selected marker genes of oxidative stress in the intestinal tissue and liver of weaning piglets fed GE compared to NC and PC. NC was set to 1.0.

Item ^4^		Diet ^1^			Sex		SEM ^2^	*p*-Value ^3^					
		NC	PC	GE	m	f		Diet	Sex	d	Diet × Sex	Diet × d	Sex × d
GPX2 (jejunum and ileum)-GPX1 (liver)													
Jejunum													
Main effects		1.00	1.26	1.16	1.00	0.93	0.2	0.45	0.89	<0.001	0.15	0.71	0.69
IA	day 27/28	1.00	1.46	1.29	1.00	1.04	0.3	0.38	0.86		0.58		
	day 55/56	1.00	1.09	1.04	1.00	0.87	0.3	0.95	0.69		0.037 *		
Ileum													
Main effects		1.00	1.01	1.06	1.00	1.01	0.1	0.87	0.97	0.71	0.85	0.30	0.13
IA	day 27/28	1.00	1.21	1.24	1.00	0.81	0.2	0.43	0.30		0.83		
	day 55/56	1.00	1.08	0.88	1.00	1.15	0.2	0.61	0.27		0.38		
Liver													
Main effects		1.00	1.12	1.15	1.00	1.05	0.1	0.26	0.48	<0.001	0.12	0.56	0.98
IA	day 27/28	1.00	1.02	1.12	1.00	1.05	0.1	0.60	0.64		0.55		
	day 55/56	1.00	1.20	1.14	1.00	1.05	0.1	0.23	0.59		0.18		
SOD1													
Jejunum													
Main effects		1.00	1.06	1.04	1.00	1.02	0.1	0.58	0.65	0.022	0.74	0.64	0.77
IA	day 27/28	1.00	1.12	1.08	1.00	1.01	0.1	0.32	0.91		0.73		
	day 55/56	1.00	1.01	1.00	1.00	1.04	0.1	0.99	0.63		0.93		
Ileum													
Main effects		1.00	0.93	0.89	1.00	0.97	0.1	0.34	0.43	0.66	0.33	0.076	0.95
IA	day 27/28	1.00	0.80	0.90	1.00	0.95	0.1	0.17	0.56		0.68		
	day 55/56	1.00	1.08	0.88	1.00	0.93	0.1	0.16	0.59		0.33		
Liver													
Main effects		1.00	1.12	1.11	1.00	1.11	0.1	0.53	0.21	<0.001	0.40	0.53	0.59
IA	day 27/28	1.00	1.02	1.13	1.00	1.06	0.1	0.62	0.61		0.84		
	day 55/56	1.00	1.19	1.08	1.00	1.16	0.1	0.46	0.21		0.41		
CAT													
Jejunum													
Main effects		1.00	0.88	0.93	1.00	1.10	0.1	0.28	0.12	0.24	0.38	0.52	0.90
IA	day 27/28	1.00	0.83	0.96	1.00	1.10	0.1	0.26	0.33		0.37		
	day 55/56	1.00	0.93	0.90	1.00	1.10	0.1	0.63	0.22		0.44		
Ileum													
Main effects		1.00	0.97	0.98	1.00	0.95	0.1	0.83	0.16	0.039	0.17	0.54	0.25
IA	day 27/28	1.00	0.97	1.03	1.00	0.99	0.1	0.55	0.82		0.16		
	day 55/56	1.00	0.84	0.92	1.00	0.88	0.1	0.73	0.13		0.059		
Liver													
Main effects		1.00	1.07	1.14	1.00	1.16	0.1	0.44	0.058	<0.001	0.51	0.88	0.41
IA	day 27/28	1.00	1.02	1.14	1.00	1.07	0.1	0.58	0.45		0.88		
	day 55/56	1.00	1.09	1.12	1.00	1.24	0.1	0.67	0.056		0.34		

GPX1/GPX2 = glutathione peroxidase; SOD1 = superoxide dismutase; CAT = catalase; ^1^ NC = negative control; PC = positive control, NC and 20 mg amoxicillin/kg BW twice a day for the first 5 days of the trial; GE = NC and grape extract, 150 g/t. ^2^ SEM: Standard error of mean based on LSMeans. ^3^ d (sampling day): day 27/28 and day 55/56 = day of the trial/post weaning. ^4^ Main effects: main effects of diet and sex = mean values at day 27/28 and day 55/56; IA: interactions: diet × d and sex × d; * Significant ANOVA with non-significant post hoc Tukey–Kramer test (*p* > 0.05).

**Table 8:** Expression of selected marker genes involved in tissue remodelling and immune activation in the intestinal tissue of weaning piglets fed GE compared to NC and PC. NC was set to 1.0.

Item ^4^		Diet ^1^			Sex		SEM ^2^	*p*-Value ^3^					
		NC	PC	GE	m	f		Diet	Sex	d	Diet × Sex	Diet × d	Sex × d
ADAM8													
Jejunum													
Main effects		1.00	1.17	1.14	1.00	1.10	0.1	0.19	0.29	0.93	0.85	0.68	0.06
IA	day 27/28	1.00	1.11	1.05	1.00	1.23	0.1	0.75	0.051		0.94		
	day 55/56	1.00	1.24	1.23	1.00	0.93	0.1	0.17	0.56		0.85		
Ileum													
Main effects		1.00	0.92	0.84	1.00	0.98	0.1	0.27	0.80	0.62	0.18	0.040	0.24
IA	day 27/28	1.00	0.73	0.86	1.00	0.90	0.2	0.20	0.38		0.73		
	day 55/56	1.00 ^ab^	1.15 ^a^	0.83 ^b^	1.00	1.09	0.1	0.036	0.42		0.11		
COL4A4													
Jejunum													
Main effects		1.00	1.00	0.85	1.00	0.93	0.2	0.66	0.86	<.0001	0.83	0.078	0.62
IA	day 27/28	1.00	0.94	0.55	1.00	1.06	0.4	0.20	0.86		0.97		
	day 55/56	1.00	1.07	1.32	1.00	0.93	0.2	0.36	0.49		0.66		
Ileum													
Main effects		1.00	1.33	0.96	1.00	1.15	0.2	0.68	0.36	0.001	0.35	0.055	0.14
IA	day 27/28	1.00	0.77	0.62	1.00	1.49	0.3	0.28	0.12		0.18		
	day 55/56	1.00	1.66	1.48	1.00	0.87	0.3	0.13	0.67		0.88		
COL7A1													
Jejunum													
Main effects		1.00	0.96	0.96	1.00	1.15	0.1	0.89	0.28	0.85	0.39	0.57	0.88
IA	day 27/28	1.00	1.06	1.04	1.00	1.07	0.2	0.93	0.54		0.45		
	day 55/56	1.00	0.87	0.89	1.00	1.10	0.1	0.51	0.36		0.52		
Ileum													
Main effects		1.00	1.09	1.10	1.00	1.05	0.1	0.43	0.38	0.54	0.53	0.93	0.15
IA	day 27/28	1.00	1.06	1.07	1.00	1.12	0.1	0.81	0.10		0.22		
	day 55/56	1.00	1.12	1.12	1.00	0.97	0.1	0.51	0.69		0.96		
LAMB3													
Jejunum													
Main effects		1.00	1.14	0.96	1.00	1.00	0.1	0.25	0.99	0.10	0.23	0.38	0.21
IA	day 27/28	1.00	1.33	1.03	1.00	0.87	0.2	0.22	0.45		0.27		
	day 55/56	1.00	0.98	0.90	1.00	1.15	0.1	0.64	0.28		0.70		
Ileum													
Main effects		1.00	1.14	1.01	1.00	0.96	0.1	0.12	0.38	0.093	0.057	0.34	0.05
IA	day 27/28	1.00	1.07	1.05	1.00	0.81	0.1	0.79	0.048		0.018		
	day 55/56	1.00 ^(ab)^	1.21 ^(a)^	0.98 ^(b)^	1.00	1.07	0.1	0.061	0.42		0.82		
SPP1													
Jejunum													
Main effects		1.00	1.18	0.98	1.00	1.07	0.2	0.67	0.81	0.020	0.85	0.19	0.12
IA	day 27/28	1.00	1.03	0.65	1.00	0.81	0.3	0.15	0.25		0.49		
	day 55/56	1.00	1.35	1.48	1.00	1.39	0.4	0.55	0.28		0.71		
Ileum													
Main effects		1.00	0.95	1.05	1.00	0.93	0.2	0.84	0.86	0.49	0.90	0.72	0.18
IA	day 27/28	1.00	1.05	1.21	1.00	0.80	0.3	0.82	0.38		0.70		
	day 55/56	1.00	0.85	0.91	1.00	1.14	0.2	0.70	0.28		0.10		

ADAM8 = ADAM metallopeptidase domain 8; COL4A4 = collagen, type IV, alpha 4; COL7A1 = collagen, type VII, alpha 1; LAMB3 = laminin, beta 3; SPP1 = secreted phosphorprotein 1. ^1^ NC = negative control; PC = positive control, NC and 20 mg amoxicillin/kg BW twice a day for the first 5 days of the trial; GE = NC and grape extract, 150 g/t. ^2^ SEM: Standard error of mean based on LSMeans. ^3^d (sampling day): day 27/28 and day 55/56 = day of the trial/post weaning. ^4^ Main effects: main effects of diet and sex = mean values at day 27/28 and day 55/56; IA: interactions: diet × d and sex × d; ^a,b^ Values within a row without a common superscript significantly differ at *p* < 0.05 (values within brackets *p* < 0.10).

**Table 9:** Expression of selected marker genes of stress involved in tissue repair and immune response in liver of weaning piglets fed GE compared to NC and PC. NC was set to 1.0.

Item ^4^		Diet ^1^			Sex		SEM ^2^	*p*-Value ^3^					
		NC	PC	GE	m	f		Diet	Sex	d	Diet × Sex	Diet × d	Sex × d
HSP70													
Main effects		1.00	1.16	1.43	1.00	1.32	0.2	0.13	0.041	0.10	0.62	0.93	0.77
IA	day 27/28	1.00	1.24	1.41	1.00	1.23	0.3	0.38	0.27		0.38		
	day 55/56	1.00	1.07	1.33	1.00	1.34	0.2	0.32	0.063		0.79		
HSP90AA1													
Main effects		1.00	0.98	0.95	1.00	1.18	0.1	0.94	0.092	0.76	0.31	0.17	0.93
IA	day 27/28	1.00	0.95	1.15	1.00	1.17	0.2	0.53	0.29		0.19		
	day 55/56	1.00	1.00	0.81	1.00	1.19	0.2	0.29	0.19		0.97		
CYP8B1													
Main effects		1.00	0.52	1.37	1.00	1.15	0.3	0.014	0.47	0.85	0.004 ^§^	0.051	0.73
IA	day 27/28	1.00 ^a^	0.24 ^b^	1.01 ^a^	1.00	1.32	0.4	<0.001	0.35		0.20		
	day 55/56	1.00	1.10	1.65	1.00	1.07	0.5	0.61	0.81		0.013 *		
MMP-13													
Main effects		1.00	0.96	1.11	1.00	1.08	0.2	0.53	0.48	0.27	0.54	0.27	0.98
IA	day 27/28	1.00	0.96	1.40	1.00	1.07	0.2	0.21	0.66		0.86		
	day 55/56	1.00	0.96	0.91	1.00	1.09	0.2	0.90	0.58		0.14		
TNFRSF14													
Main effects		1.00	0.81	1.01	1.00	0.93	0.2	0.28	0.54	0.071	0.88	0.43	0.38
IA	day 27/28	1.00	0.65	0.97	1.00	0.81	0.2	0.17	0.32		0.36		
	day 55/56	1.00	0.98	1.04	1.00	1.01	0.2	0.95	0.85		0.67		
CCL4													
Main effects		1.00	0.94	1.14	1.00	0.98	0.1	0.16	0.80	<0.001	0.35	0.26	0.53
IA	day 27/28	1.00	0.81	1.15	1.00	0.93	0.2	0.12	0.59		0.082		
	day 55/56	1.00	1.06	1.10	1.00	1.05	0.1	0.73	0.75		0.66		

HSP70 = heat shock protein; HSP90AA1 = 90 kDa heat shock protein; CYP8B1 = cytochrome *p*-450 8B1; MMP-13 = matrix metalloproteinase 13 precursor; TNFRSF14 = tumour necrosis factor receptor superfamily, member 14; CCL4 = chemokine (C–C motif) ligand 4; ^1^ NC = negative control; PC = positive control, NC and 20 mg amoxicillin/kg BW twice a day for the first 5 days of the trial; GE = NC and grape extract, 150 g/t. ^2^ SEM: Standard error of mean based on LSMeans. ^3^ d (sampling day): day 27/28 and day 55/56 = day of the trial/post weaning. ^4^ Main effects: main effects of diet and sex = mean values at day 27/28 and day 55/56; IA: interactions: diet × d and sex × d; * Significant ANOVA with non-significant multiple pairwise comparisons (*p* > 0.05). ^§^ Details of the interaction are shown in Supplementary Materials, Table S4.

**Table 10:** Plasma antioxidant measurements and selected acute-phase proteins (APPs) of weaning piglets fed GE compared to NC and PC.

Item ^4^		Diet ^1^			Sex		SEM ^2^	*p*-Value ^3^					
		NC	PC	GE	m	f		Diet	Sex	d	Diet × Sex	Diet × d	Sex × d
Antioxidant measurements													
SOD (U/ml)													
Main effects		5.09	5.12	4.71	5.14	4.81	0.32	0.63	0.38	0.014	0.86	0.89	0.65
IA	day 0	5.96	5.74	5.35	6.13	5.24	0.58	0.78	0.22		0.98		
	day 6	4.75	4.58	4.85	4.90	4.55	0.41	0.89	0.45		0.79		
	day 27/28	4.23	4.65	3.50	4.00	4.26	0.52	0.26	0.63		0.56		
	day 55/56	5.41	5.58	5.20	5.58	5.23	0.73	0.93	0.66		0.89		
MDA (µM)													
Main effects		2.31	2.25	2.04	2.13	2.26	0.26	0.77	0.68	<0.001	0.89	0.11	0.83
IA	day 0	3.24	3.64	3.52	3.31	3.60	0.37	0.74	0.47		0.18		
	day 6	1.62 ^(ab)^	2.56 ^(a)^	1.52 ^(b)^	1.78	2.02	0.31	0.041*	0.50		0.46		
	day 27/28	1.37	1.08	1.32	0.98	1.54	0.30	0.79	0.13		0.52		
	day 55/56	2.96	2.04	1.65	2.46	1.98	0.52	0.19	0.43		0.34		
Acute-phase proteins													
Haptoglobin (µg/mL)													
Main effects		769	676	720	634	809	93	0.78	0.11	0.070	0.10	0.86	0.07
IA	day 6	957	808	805	802	911	126	0.62	0.46		0.78		
	day 27/28	661	619	646	695	589	161	0.98	0.58		0.074		
	day 55/56	690	598	695	402	920	136	0.83	0.002		0.52		
pigMAP (µg/mL)													
Main effects		794	757	797	790	774	48	0.81	0.77	<0.001	0.27	0.63	0.74
IA	day 6	1167	1221	1272	1253	1187	82	0.67	0.49		0.27		
	day 27/28	572	531	549	552	549	39	0.76	0.95		0.39		
	day 55/56	645	518	577	571	589	46	0.14	0.74		0.080		

SOD = superoxide dismutase; MDA = malondialdehyde; pigMAP = major acute-phase protein. ^1^ NC = negative control; PC = positive control, NC and 20 mg amoxicillin/kg BW twice a day for the first 5 days of the trial; GE = NC and grape extract, 150 g/t. ^2^ SEM: Standard error of mean based on LSMeans. ^3^ d (sampling day): day 0, day 6, day 27/28, and day 55/56 = day of the trial/post weaning. ^4^ Main effects: main effects of diet and sex = mean values at day 0, day 6, day 27/28 and day 55/56; IA: interactions: diet × d and sex × d; ^a,b^ Values within a row without a common superscript significantly differ at *p* ≤ 0.05 (values within brackets *p* < 0.10). * Significant ANOVA (*p* < 0.05) and trend (*p* < 0.10) in multiple pairwise comparisons.

## Data Availability

The datasets supporting the conclusions of this article are included within the article, as well within the Supplementary Materials.

## Supplementary Materials

The following supporting information can be downloaded at: https://www.mdpi.com/article/10.3390/antiox11081428/s1, Table S1: Interactions between factors diet and sex detected for parameter villus height in jejunum of weaning piglets; Table S2: Interactions between factors diet and sex detected for parameter villus surface in jejunum of weaning piglets; Table S3: Effect of dietary GE supplementation on the thickness of intestinal muscular layer of weaning piglets compared to NC and PC; Table S4: Interactions between factors diet and sex detected for expression of gene CYP8B1 in liver of weaning piglets. NC males were set to 1.0.
