# Supplementary material for: Evaluation of a Dietary Grape Extract on Oxidative Status, Intestinal Morphology, Plasma Acute-Phase Proteins and Inflammation Parameters of Weaning Piglets at Various Points of Time
Source: Antioxidants (Basel). 2022 Jul 22;11(8):1428. doi: 10.3390/antiox11081428 (PMC9394324; doi:10.3390/antiox11081428)
Supplement: Supplementary file 1 [file antioxidants-11-01428-s001.zip › antioxidants-1793627-supplementary.pdf]

Table S1. Interactions between factors diet and sex detected for parameter villus height in jejunum of weaning piglets.

	Villus height (μm) (SEM = 13.2)		
Main effect	Diet		
Sex	NC	PC	GE
m	256 [b]	258 [b]	333 [a]
f	291	283	289
p-value			
Diet	0.005		
Sex	0.60		
Diet x sex	0.008		

SEM = Standard error of mean based on LSMeans; NC = negative control; PC = positive control, NC+ 20 mg amoxicillin/kg BW twice a day for the first 5 days of the trial; GE = NC + grape extract, 150 g/t; [a,b] Values within a row without a common superscript differ significantly at $p < 0.05$.

Table S2. Interactions between factors diet and sex detected for parameter villus surface in jejunum of weaning piglets.

	Villus surface (μm) (SEM = 2.2)		
Main effect	Diet		
Sex	NC	PC	GE
m	29.5 [b]	31.1 [b]	41.6 [a]
f	34.0	34.8	33.4
p-value			
Diet	0.023		
Sex	0.98		
Diet x sex	0.006		

SEM = Standard error of mean based on LSMeans; NC = negative control; PC = positive control, NC+ 20 mg amoxicillin/kg BW twice a day for the first 5 days of the trial; GE = NC + grape extract, 150 g/t; [a,b] Values within a row without a common superscript differ significantly at $p < 0.05$.

Table S3. Effect of dietary GE supplementation on the thickness of intestinal muscular layer of weaning piglets, compared to NC and PC.

Item [4]		Diet [1]			Sex		SEM [2]	p-value [3]					
		NC	PC	GE	m	f		diet	sex	d	diet × sex	diet × d	sex × d
Tunica muscularis thickness (μm)													
Jejunum													
	Main effects	258	271	280	274	265	7.7	0.13	0.30	<.001	0.065	0.80	0.80
Interactions	day 27/28	241	248	256	254	242	12	0.69	0.40		0.021 *		
Interactions	day 55/56	274	293	306	293	288	16	0.37	0.78		0.35		
Ileum													
	Main effects	478	475	475	505	447	26	0.99	0.054	<0.001	0.19	0.71	0.52
Interactions	day 27/28	358	356	382	385	346	32	0.82	0.30		0.94		
Interactions	day 55/56	598	593	568	625	548	40	0.85	0.11		0.13		
Colon													
	Main effects	372	351	331	344	359	19	0.32	0.51	<0.001	0.47	0.53	0.90
Interactions	day 27/28	335	293	303	305	315	25	0.45	0.72		0.61		
Interactions	day 55/56	411	409	359	384	401	30	0.38	0.62		0.71		

[1] NC = negative control; PC = positive control, NC+ 20 mg amoxicillin/kg BW twice a day for the first 5 days of the feeding trial; GE = NC + grape extract, 150 g/t. [2] SEM Standard error of mean based on LSMeans. [3] d (sampling day): day 27/28, day 55/56 = day of the trial/post weaning. [4] Main effect: main effect diet and sex, respectively = mean of the values at day 27/28 and day 55/56; Interactions: diet x d and sex x d, respectively. * Significant ANOVA with non-significant post hoc Tukey-Kramer Test ($p > 0.05$).

Table S4. Interactions between factors diet and sex detected for expression of gene CYP8B1 in liver of weaning piglets. NC males were set to 1.0.

	CYP8B1 (SEM = 0.5)		
Main effect	Diet		
Sex	NC	PC	GE
m	1.00 ^{b}	6.06 ^{ax}	1.15 ^{b}
f	2.30	1.52 ^{y}	1.23
p-value			
Diet	0.014		
Sex	0.47		
Diet x sex	0.004		

SEM = Standard error of mean based on LSMeans; NC = negative control; PC = positive control, NC+ 20 mg amoxicillin/kg BW twice a day for the first 5 days of the trial; GE = NC + grape extract, 150 g/t; ^{a,b} Values within a row without a common superscript differ significantly at $p < 0.05$. ^{x,y} Values within a column without a common superscript differ significantly at $p < 0.05$.
